# Advancements in Gellan Gum-Based Films and Coatings for Active and Intelligent Packaging

**DOI:** 10.3390/polym16172402

**Published:** 2024-08-24

**Authors:** Hang Li, Kun Gao, Huan Guo, Rongfeng Li, Guantian Li

**Affiliations:** 1 CAS and Shandong Province Key Laboratory of Experimental Marine Biology, Center for Ocean Mega-Science, Institute of Oceanology, Chinese Academy of Sciences, 7 Nanhai Road, Qingdao 266071, China; 2 Laboratory for Marine Biology and Biotechnology, Qingdao Marine Science and Technology Center, Qingdao 266237, China; 3 College of Biomass Science and Engineering and Healthy Food Evaluation Research Center, Sichuan University, Chengdu 610065, China; 4 Laboratory for Marine Drugs and Bioproducts, Qingdao Marine Science and Technology Center, Qingdao 266237, China

**Keywords:** biodegradable, food preservation, natural compound, encapsulation, carbohydrate

## Abstract

Gellan gum (GG) is a natural polysaccharide with a wide range of industrial applications. This review aims to investigate the potential of GG-based films and coatings to act as environmentally friendly substitutes for traditional petrochemical plastics in food packaging. GG-based films and coatings exhibit versatile properties that can be tailored through the incorporation of various substances, such as plant extracts, microorganisms, and nanoparticles. These functional additives enhance properties like the light barrier, antioxidant activity, and antimicrobial capabilities, all of which are essential for extending the shelf-life of perishable food items. The ability to control the release of active compounds, along with the adaptability of GG-based films and coatings to different food products, highlights their effectiveness in preserving quality and inhibiting microbial growth. Furthermore, GG-based composites that incorporate natural pigments can serve as visual indicators for monitoring food freshness. Overall, GG-based composites present a promising avenue for the development of sustainable and innovative food packaging solutions.

## 1. Introduction

The global demand for sustainable and eco-friendly packaging solutions has led to an increased interest in polysaccharide-based materials [1]. As the world grapples with the environmental impacts of plastic pollution [2], polysaccharides offer a biodegradable and renewable alternative in terms of packaging applications [1]. Polysaccharides, such as pectin [3], chitosan [4], starch [5], konjac glucomannan [6], and gellan gum (GG) [7], are derived from natural sources and are known for their excellent film-forming capabilities. These biopolymers are not only abundant and inexpensive but also possess unique physicochemical properties that make them suitable for a wide range of applications. Polysaccharide-based packaging materials are particularly appealing because they can be engineered to provide desirable barrier properties, mechanical strength, edibility, and compatibility with food products [8,9,10]. Moreover, the biodegradability of these materials ensures a minimal environmental impact [11,12,13], addressing the critical issue of plastic waste in landfills and oceans. As a result, polysaccharide packaging materials are increasingly being explored as sustainable alternatives to conventional petroleum-based plastics in various industries.

**Figure 1:**
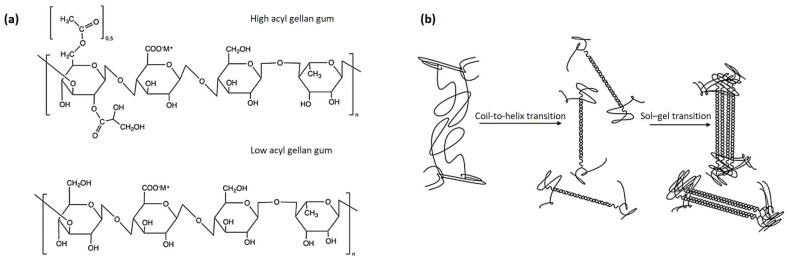
Structure of gellan gum [15]. (**a**) High- and low-acyl gellan gum. (**b**) Coil-to-helix and sol-gel transition of gellan gum. Reprinted with permission from Elsevier.

**Figure 2:**
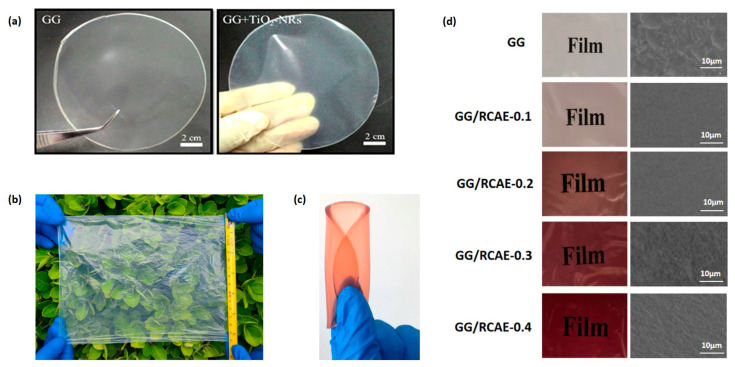
Appearance of gellan gum-based films. (**a**) Gellan gum film containing titanium dioxide nanoparticles [35]. (**b**) Gellan gum/cellulose/chitosan composite film [32]. (**c**) Gellan/gelatin film containing red radish anthocyanins [44]. (**d**) Gellan gum film incorporating red cabbage anthocyanins [43]. (**a**) was used under a CC-BY 4.0 license. (**b**,**c**) were reprinted with permission from the American Chemical Society. (**d**) was reprinted with permission from Elsevier.

GG is a high-molecular-weight, water-soluble polysaccharide produced by the bacterium *Sphingomonas* sp. [14]. It is composed of a repeating tetrasaccharide unit containing 1,3-β-d-glucose, 1,4-β-d-glucuronic acid, 1,4-β-d-glucose, and 1,4-α-l-rhamnose (Figure 1). GG molecules spontaneously assemble into a double helix conformation [15]. These double helices further aggregate to create a three-dimensional network structure in suitable aqueous conditions (e.g., with the appropriate pH and temperature) [15]. The versatility of GG has led to its widespread use in various industries. In the biomedical sector, it is utilized in drug delivery systems [16], tissue engineering scaffolds [17], and wound dressing [18] due to its biocompatibility and non-toxic nature. In the food industry, GG serves as a thickening, stabilizing, and gelling agent in products such as confectionery [19], dairy products [20], and beverages [21]. The unique properties and multifunctionality of GG make it a promising material in developing advanced packaging solutions that cater to both functional and sustainability requirements. GG has received official authorization for use in numerous countries. The Food and Drug Administration of United States has approved GG as a direct food additive, considering it “generally recognized as safe (GRAS)” for various applications in food products (21 CFR § 172.665) [22]. The European Food Safety Authority has also evaluated GG and approved its use as a food additive (E 418) in the European Union [23]. China has approved GG for use in food products based on its National Food Safety Standard for Food Additives (GB 25535-2010) [24]. Similarly, Health Canada has authorized the use of GG in various food categories [25]. These regulatory approvals across multiple jurisdictions underscore the safety and acceptability of GG as a food ingredient and packaging material. Particularly, GG can also be used as a packaging material (Figure 2). These polymers can extend the shelf-life of different food products, such as pork [26], fish [27], shrimp [28], dairy products [29], fruit [30], and vegetable [31]. The protective effects of GG-based composites on certain food products are even better than those of polyethylene [9,31,32] and polypropylene [13]. The growing demand for biodegradable food packaging materials, driven by increasing environmental concerns and stricter regulations, has further intensified the interest in GG. As a natural, renewable, and biodegradable polymer, GG aligns with the principles of a circular economy and offers a viable solution with which to mitigate the environmental impact of plastic waste.

While GG shows great potential in developing biodegradable packaging materials, there are inherent challenges that could limit its broader application. One significant issue is the limited antioxidant capacity of neat GG composites [31], which may leave food products prone to oxidative degradation. Furthermore, these composites do not provide adequate protection against ultraviolet (UV) light [11,33], potentially hastening the spoilage of light-sensitive products. Additionally, the antimicrobial properties of GG composites are often insufficient [34,35,36], which can increase the risk of microbial contamination in packaged foods. To overcome these drawbacks, the integration of various functional compounds into GG-based composites is frequently necessary in order to boost their protective properties and broaden their application scope. These enhancements often include the addition of polyphenols [26,37], essential oils [9,38,39], beneficial microorganisms [30,40], metal oxides [33,41], and food preservatives [29,42]. Additionally, the use of natural pigments such as anthocyanins, which are derived from the vibrant colors of many plant organs, has become increasingly popular [43,44]. These pigments are particularly valued for their pH-sensitive color changes, serving as effective indicators of food freshness in GG-based packaging solutions.

**Figure 3:**
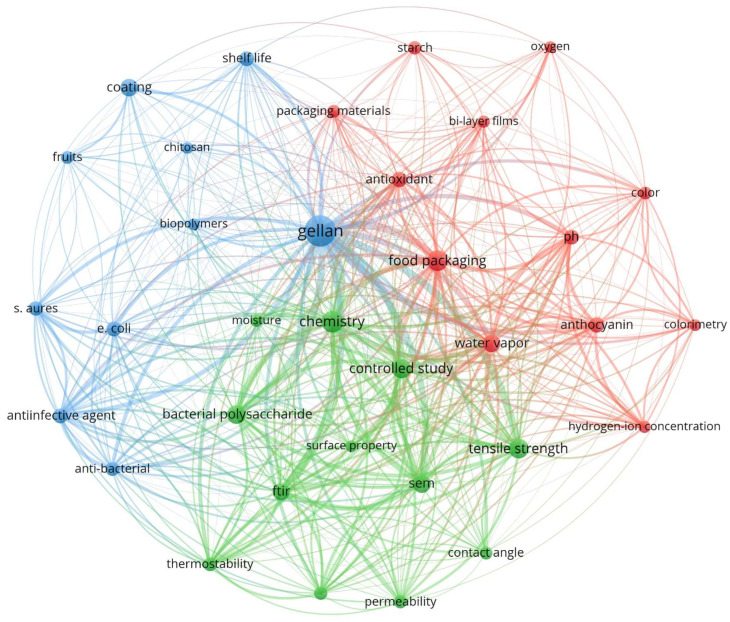
Bibliometric network map based on the co-occurrence of terms in recent studies (2015–2024) on gellan gum-based packaging materials.

In recent years, GG-based materials have garnered significant interest as potential candidates for food packaging applications. To better understand the latest developments and trends in this field, a bibliometric analysis of relevant publications was undertaken. Scopus was used as the primary database, with articles published between 2015 and 2024 identified by using the query string “TITLE-ABS-KEY (gellan AND (film OR coating)) AND PUBYEAR > 2015 AND PUBYEAR < 2025” as the search criteria. The resulting dataset was then subjected to analysis using VOSviewer (version 1.6.20) [45]. The tool facilitates a comprehensive bibliometric evaluation, providing insights into the evolving landscape of GG-based packaging materials research (Figure 3). The term “gellan” is positioned at the center of the network, indicating its role as the primary focus of the research and publications. The connections radiating from GG to various topics highlight its integral role in multiple aspects of packaging technology. The map is divided into distinct clusters, each representing a thematic area within GG-based packaging research. The green cluster explores the structural and mechanical aspects of GG-based packaging materials. The properties of tensile strength, permeability, and thermostability are crucial for determining the durability and effectiveness of the films in packaging applications. The red cluster emphasizes the use of GG films as intelligent packaging solutions capable of monitoring food quality. The inclusion of colorimetric indicators like anthocyanins enables pH-sensitive responses that help to track spoilage through visual changes. Additionally, the focus on the barrier properties of GG composites, such as water vapor permeability, underscores their ability to maintain food quality by controlling moisture and oxygen exchange. The blue cluster focuses on the antimicrobial properties and applications of GG-based composites, particularly their effectiveness against common pathogens like *Staphylococcus aureus* and *Escherichia coli*. The integration of substances such as antimicrobials enhances the protective qualities of GG composites, making them suitable for extending the shelf-life of fruits and other perishable items. In conclusion, the bibliometric analysis highlights GG-based packaging materials as a promising area of research focused on improving food safety, extending shelf-life, and providing environmentally sustainable alternatives to traditional packaging.

While the biomedical applications of GG have been extensively explored and reviewed [14,46,47], there remains a significant gap in the literature regarding the development and potential of GG-based composites for use in active and intelligent packaging. This manuscript presents a comprehensive study on the development of GG-based active and intelligent packaging films with enhanced functional properties. The research focuses on the incorporation of various functional additives, including essential oils, polyphenols, and nanoparticles, to improve the mechanical, barrier, antioxidant, and antimicrobial properties of GG-based composites. Furthermore, this study explores the integration of colorimetric indicators into GG films, allowing for the real-time monitoring of food freshness by examining visible color changes in response to pH variations. The environmental impact of the GG-based films is also assessed, emphasizing their biodegradability and potential as sustainable packaging alternatives. This work aims to demonstrate the feasibility and effectiveness of GG-based films as intelligent packaging solutions, offering innovative approaches with which to improve food safety, extend shelf-life, and reduce environmental impact. By leveraging the unique properties of GG and the functionalities of incorporated materials, this research contributes to the development of sustainable and high-performance packaging technologies that meet the evolving demands of consumers and the food industry.

## 2. Preparation of GG-Based Films and Coatings

### 2.1. Gel Matrix Materials

GG can be utilized as the primary component in the development of packaging [30,43,48,49]. However, the effectiveness of GG-based films and coatings is often enhanced by incorporating a variety of base matrix materials, which contribute unique properties that improve the overall functionality of the composites. Among these, polysaccharides are frequently employed due to their beneficial characteristics. Notable examples include agar [50,51,52], alginate [53,54], carrageenan [55,56,57], cellulose [32,53], chitosan [38,58], flaxseed gel [59], guar gum [60,61], konjac glucomannan [55,62,63], levan [12], pectin [64,65], and starch [66,67,68,69]. Furthermore, to diversify the properties of GG-based films, proteins are often integrated into the gel matrices. Commonly used proteins include gelatin [40], lactoferrin [70,71], pea protein [72], and soy protein [73]. In addition to polysaccharides and proteins, degradable polymers like polyvinyl alcohol [9,74,75] and polyester [10,26,76] are also blended with GG to form gel matrices. The selection of specific gel matrix materials should be guided by the desired characteristics of the final product, ensuring that the films or coatings meet the required performance standards for specific applications.

### 2.2. Functional Compounds

#### 2.2.1. Functional Compounds for Active Packaging

Neat GG composites usually show limited functionalities, such as weak UV shielding [11,33,77] and antimicrobial [34,35,78] capabilities. The addition of functional compounds into GG-based composites can improve their functionalities, thereby enhancing the shelf-life and quality of packaged food.

Essential oils are frequently incorporated into polysaccharide-based packaging materials to enhance their functionality [79]. The essential oils used in GG-based composites include basil [80], clove [64], dill [13], fennel [54], mustard [38], myrtle [9], oregano [39], rosemary [78,81], and thyme [58,82] essential oils. Geraniol, a monoterpenoid widely found in certain essential oils, is also used in GG composites [83]. Thymol, the monoterpenoid found in thyme essential oil, also enhances the protective effects of GG composites [63,84]. Other essential oils, such as ginger, garlic, lemon, lemongrass, orange peel, and peppermint essential oils, remain to be studied with GG composites.

Polyphenols are also often integrated into polysaccharide-based composites [1]. Phenolic compounds can be added in the form of isolates, such as dihydromyricetin [85], ferulic acid [10], gallic acid [37], *p*-coumaric [26], and vanillin [83], and polyphenol-rich extracts such as coffee parchment waste extract [36] and pomegranate peel extract [86].

Beyond the plant-derived natural compounds mentioned above, nanoparticles can be used in polysaccharide packaging [87]. Various nanoparticles are incorporated into GG composites. These include metal nanoparticles such as copper oxide (CuO) [31], silver nanoparticles [88], titanium oxide (TiO_2_) [31,51], and zinc oxide (ZnO) [11,33,41]. Other nanoparticles used in previous studies also include silicon dioxide (SiO_2_) [89], cellulose nanocrystals [50,90,91], nanochitosan [70], and eggshell nanoparticles [48]. Nanoclays, carbon dots, and graphene have not been used in recent studies as additives in GG-based packaging materials, and this can be studied in the future.

Probiotic strains like *Bifidobacterium longum* [40] and *Lactococcus lactis* [30] have been used in GG composites. Other probiotic strains have not been studied for use with GG composites in packaging materials. In addition, bacteriophages may also be used in GG composites to enhance antimicrobial capabilities [92]. The supernatants of *Streptococcus infantarius* cultures enhance the protective effects of GG composites, probably owing to the introduction of the antimicrobial metabolites of the strain [27,42]. The direct addition of microbial metabolites like natamycin [64] and nisin [60,65,67] improves the protective effects of GG composites.

There are other kinds of functional compounds that have been used in GG-based composites to enhance functionality. An example is ethylene diamine tetraacetic acid (EDTA), the compound usually used as a food presentive or stabilizer [42]. Potassium sorbate is also usually used as a food presentive [29]. These food additives have been used in the GG-based composites. This demonstrates the wide range of functional compounds that can be utilized to tailor the properties of GG-based materials for various applications.

Overall, the incorporation of diverse functional compounds into GG composites offers a promising avenue for developing advanced packaging materials with enhanced properties. However, it is important to consider the potential interactions between different additives and their impact on the overall performance and safety of the final product. Further research is needed to explore the synergistic effects of various combinations of functional compounds and to optimize their concentrations for specific applications.

#### 2.2.2. Functional Compounds for Intelligent Packaging

Anthocyanins, a key subgroup within the polyphenol family, are widely utilized in polysaccharide-based packaging. While the addition of these compounds can extend the shelf-life of food products, they are also used as pH-sensing indicators due to their colorimetric changes during food spoilage. The versatility of anthocyanins is further demonstrated by the diverse range of sources utilized in previous studies. These include colored vegetables, fruits, seeds, and petals, such as black rice [41,49,93], blueberry [40], *Broussonetia papyrifera* fruit [55], *Clitoria ternatea* flower [73,90], dragon fruit pericarp [94], elderberry [95], mulberry [33,96], purple kale [94], purple sweet potato [97], red cabbage [43], red radish [44], rose [51], and roselle [67]. In addition to anthocyanins, other polyphenols like curcumin have also been incorporated into GG films for pH-sensing purposes [41,49]. Synthetic chemicals such as methyl red and bromothymol blue have been employed in GG-based films to provide visual indicators of food quality, reflecting changes in the packaging environment [53].

### 2.3. General Steps to Prepare GG-Based Films and Coatings

#### 2.3.1. Preparation of Film-Forming Solutions

**Figure 4:**
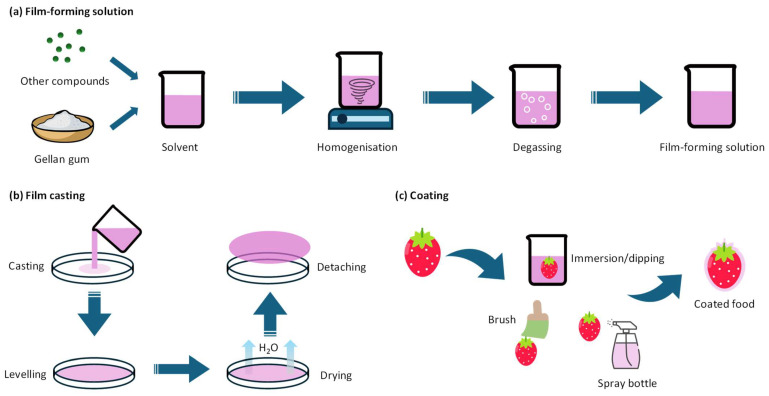
Methods to prepare gellan gum-based films and coatings. (**a**) Preparation of film-forming solution. (**b**) Casting method to prepare film. (**c**) Methods to deposit coating onto the food surface.

The creation of GG-based films and coatings generally involves two main steps: preparing the film-forming solution (Figure 4a) and producing the film or coating itself (Figure 4b,c). The initial step entails dispersing and homogenizing GG, a plasticizer (often glycerol), and potentially other functional or film-forming compounds in a solvent, typically water [41]. Critically, a degassing step follows to remove any trapped air bubbles that could create voids in the final product. This can be achieved through sonication [33,53] and constant stirring [70,80]. Treatments like vacuum and centrifugation may also be applicable to GG solutions. The choice of degassing technique may depend on various factors, including the viscosity, the presence of sensitive components, and the production scale.

#### 2.3.2. Fabrication of GG-Based Films

Casting is the most prevalent method for fabricating GG-based films (Figure 4b) (Table 1). This technique involves pouring the pre-prepared, degassed film-forming mixture into a mold (e.g., Petri dish or Teflon plate), followed by controlled drying in an oven or incubator at varying temperatures [58,72,74]. The resulting film can then be easily peeled off the mold. Casting is favored for its simplicity and scalability, making it applicable in both laboratory research and large-scale industrial production. However, while casting remains the most common and accessible method, the exploration of alternative techniques like electrospinning, extrusion, and 3D printing indicates ongoing efforts to diversify the production capabilities of GG-based films for food preservation.

#### 2.3.3. Fabrication of GG-Based Coatings

Degassed GG-based solutions can be directly used to apply coatings onto the external surfaces of food items. There are three commonly used methods for applying these coatings: immersion, brushing, and spray coating (Figure 4c). In immersion coating, the food is directly dipped into the film-forming solution [59,83]. This technique is particularly effective for smaller or intricately shaped products, ensuring even coverage and penetration of the coating into crevices. Brushing involves the application of the film-forming solution onto the food surface using a brush [80]. While less commonly reported in the previously published literature, it offers a simple and direct approach to coating application. Spray coating, on the other hand, involves atomizing the solution and spraying it onto the food surface, providing more control over the thickness and uniformity of the coating [91], which is advantageous for larger-scale operations or simpler product shapes. The choice between these methods depends on factors such as the size and shape of the food products, the thickness desired for the coating, and the equipment available. After application, the coated food products usually undergo a drying process at room temperature or within a controlled environment [91]. This step is critical for maintaining the integrity of the coating and preventing any microbial growth that could result from residual moisture.

#### 2.3.4. Fabrication of Multi-Layered Films and Coatings

Bilayer [33,53] or triple-layer [58,74] GG films can be fabricated. The fabrication of multi-layered GG-based films and coatings involves the sequential application of multiple layers, each with a distinct composition. This is achieved by repeating the steps used for single-layered coatings, starting with a base layer of GG composite, followed by the deposition of additional layers with varying compositions [53,96]. Alternatively, individual layers can be sequentially subjected to compression molding using a hydraulic press, resulting in a unified, multi-layered structure [10,76]. Multi-layered films enable the incorporation of various functional compounds within each layer, tailoring the overall properties of the composite to meet specific needs. For example, one layer might focus on pH sensing for freshness indication, while another could provide antimicrobial protection [33,53]. In addition to the multi-layered films, the multi-layered coatings may simply be fabricated by dispensing a layer of film-forming solution onto the surface of food products sequentially [59]. Research has shown that multi-layered composites can outperform single-layered counterparts in various aspects, such as mechanical properties and antimicrobial effects [41]. The capacity to incorporate various functional compounds within separate layers offers the flexibility to design coatings with complex, tailored properties that enhance the protective effectiveness of the packaging.

## 3. Physicochemical Properties of GG-Based Composites

### 3.1. Mechanical Properties

The mechanical properties of GG films, specifically tensile strength and elongation at break, are crucial indicators of their suitability for various applications, especially in food packaging, where durability and flexibility are essential. The incorporation of various additives into GG films can significantly influence these properties, enhancing their functionality and application scope (Table 2).

The addition of xanthan gum to GG composites generally leads to a decrease in tensile strength beyond a certain concentration [98]. A GG/xanthan gum ratio of 7:3 maximizes tensile strength at 68.0 MPa, but higher xanthan concentrations reduce this strength to 40.2 MPa, demonstrating the delicate balance needed in composite formulations [98]. Incorporating a second layer, such as zein, into GG films increases tensile strength while decreasing elongation at break [93]. This indicates that layering can enhance the mechanical properties of GG films, potentially creating a more robust and cohesive structure.

The addition of inorganic nanoparticles to GG composites also influenced their mechanical properties. The addition of 1–7% TiO_2_ nanoparticles increased tensile strength (from 48.2 to 56.1 MPa) but decreased the elongation at break (from 28.2% to 23.0%) of GG/xanthan gum/carrageenan films [34]. Similarly, the addition of 1–5% ZnO nanoparticles improved tensile strength in GG/xanthan gum films (from 22.1 to 35.5 MPa) [77], probably attributable to the intermolecular crosslinking effect of ZnO. Incorporating 5% SiO_2_ nanoparticles into GG/cellulose films also enhanced their tensile strength, from 28.2 to 45.1 MPa [89]. The strengthened structure of GG composites with the addition of these nanoparticles may explain the enhanced tensile strength.

The addition of black rice anthocyanins (4–12%) to GG/zein films increased tensile strength (from 7.58 to 8.91 MPa) but decreased the elongation at break (from 7.68% to 5.37%) [93]. This was likely due to the hydrophilic nature of anthocyanins in the extract, which can form hydrogen bonds with water molecules, making the films stiffer and less extensible. However, the addition of elderberry anthocyanins (4–12%) to GG/gelatin films significantly increased tensile strength (from 5.46 to 14.57 MPa) without affecting elongation at break [95]. In addition, the inclusion of *Clitoria ternatea* anthocyanins (0.6 mg/mL) in GG films did not affect the tensile strength and elongation at break [73]. These results suggest that the specific type of anthocyanin and its interaction with the film matrix play a critical role in determining film mechanical properties. Similarly, the effects of essential oils on mechanical properties vary. While rosemary essential oil (5–30%) decreased both tensile strength and the elongation at break in GG/cellulose films [78], dill essential oil (4–4%) increased tensile strength while decreasing the elongation at break in similar films [13]. These contrasting effects highlight the complex interactions between essential oils and GG polymer matrices, which can either reinforce or weaken the structural integrity of films.

In conclusion, the mechanical properties of GG-based films are highly tunable through the incorporation of various additives. Understanding the complex interplay between these components is critical for designing films with desired mechanical properties for specific applications. Future research should focus on elucidating the mechanisms behind these interactions and exploring novel additives to further expand the range of mechanical properties achievable with GG-based films.

### 3.2. Water Barrier Properties

#### 3.2.1. Water Vapor Permeability

Water vapor permeability (WVP) is a critical parameter in food packaging, influencing the shelf-life and quality of food products by controlling moisture transfer. Modifying the WVP of GG-based films can either prevent moisture ingress, extending shelf-life, or allow for controlled moisture release in breathable packaging.

The composition and structural design of GG films impact their WVP. For example, the incorporation of guar gum (20–40%) into GG films led to an increase in WVP (from 1.18 × 10^−5^ to 1.60 × 10^−5^ g·mm^−1^·s^−1^·Pa^−1^), attributed to the hydrophilic nature of guar gum [60]. Combining GG with xanthan gum at a 7:3 ratio resulted in the lowest WVP (4.18 × 10^−13^ g·cm^−1^·s^−1^·Pa^−1^) among the different mixing ratios tested [98]. GG/zein bilayer films (4.89 × 10^−10^ g·m^−1^·s^−1^·Pa^−1^) exhibited lower WVP than the single-layer GG film (5.33 × 10^−10^ g·m^−1^·s^−1^·Pa^−1^) [93]. The WVP of roselle anthocyanin-incorporated single-layer GG film was 6.45 × 10^−11^ g·m^−1^·s^−1^·Pa^−1^; adding an extra layer of nisin-incorporated cellulose/starch composite to the film decreased the WVP to 5.52 × 10^−11^ g·m^−1^·s^−1^·Pa^−1^ [67]. The incorporation of *Broussonetia papyrifera* fruit anthocyanin and mica nanosheets into a double-layer GG film significantly reduced WVP compared to neat single-layer films [55]. This reduction is attributed to the tortuous path created by these fillers, hindering water vapor diffusion.

The addition of metal nanoparticles has been shown to decrease the WVP of GG-based films. For instance, 1–5% ZnO nanoparticles reduced the WVP of GG film from 2.20 × 10^−9^ to 1.60 × 10^−9^ g·m^−2^·s^−1^·Pa^−1^ [11]. Similarly, the addition of 1–5% ZnO nanoparticles decreased the WVP of GG/xanthan gum films from 3.83 × 10^−9^ to 2.31 × 10^−9^ g·m^−2^·s^−1^·Pa^−1^ [77]. In addition, the incorporation of 1–7% TiO_2_ into GG/xanthan gum/carrageenan films decreased the WVP from 1.80 × 10^−9^ to 11.58 × 10^−9^ g·m^−2^·s^−1^·Pa^−1^ [34]. These observations highlight the ability of metal nanoparticles to tighten the film matrix and reduce micro-paths within the composite structure.

Essential oils, known for their hydrophobicity, generally influence WVP in GG films. For example, increasing thyme essential oil content increased the WVP of GG/cellulose films, from 9.58 × 10^−11^ to 19.19 × 10^−11^ g·m^−1^·s^−1^·Pa^−1^ [58], potentially due to the disruption of film integrity. Similarly, increasing rosemary essential oil concentration (5–30%) led to higher WVP in GG/cellulose films [78]. On the other hand, the addition of 2–4% of dill essential oil did not affect the WVP of GG/cellulose film [13]. The discrepant effects of essential oils from these studies suggest the complex interaction between essential oils and polymer matrices.

Other functional compounds also affect the WVP of GG-based composites. EDTA incorporation significantly reduced the WVP of GG films by ~85%, possibly due to structural modifications caused by EDTA–calcium crystal aggregates [42]. The addition of flavanonol dihydromyricetin to GG/konjac glucomannan films decreased WVP from 6.46 to 4.03 g·mm·m^−2^·d^−1^·kPa^−1^ [85]. Conversely, the addition of soy protein to GG films increased WVP, likely due to the disruption of the GG network by the protein [73]. However, adding *Clitoria ternatea* anthocyanin did not affect the WVP of GG/soy protein composites [73]. In addition, it was also found that the addition of nisin to GG/pectin films did not affect WVP [65].

These diverse findings highlight the complex relationship between GG film composition and WVP. Further research is needed to elucidate the mechanisms underlying the observed effects of different additives and to develop GG-based films with tailored WVP properties for specific applications. Understanding these mechanisms will enable the design of films that can optimally control moisture transfer, thereby enhancing the shelf-life and quality of packaged food products.

#### 3.2.2. Contact Angle

**Figure 5:**
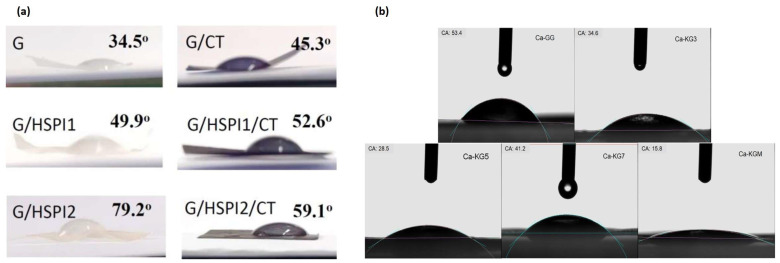
Contact angle of gellan gum-based composites. (**a**) Gellan gum/soy protein composites with *Clitoria ternatea* flower extracts [73]. (**b**) Gellan gum/konjac glucomannan composites with gallic acids [37]. Both figures were reprinted with permission from Elsevier.

The contact angle of GG-based films, reflecting their surface hydrophobicity, is an important parameter influencing moisture penetration and overall film performance. Research indicates that incorporating various additives can significantly alter the contact angle of these films (Figure 5). Adding TiO_2_ nanoparticles to GG/xanthan gum/carrageenan films resulted in a notable increase in water contact angle from 84.2° to 111.3° [34]. Similarly, ZnO nanoparticles enhanced the contact angle of GG/xanthan gum films [77] and GG composite films [11], primarily due to the hydrophobic nature of ZnO. As for the incorporation of essential oil, incorporating rosemary essential oil (5–20%) into GG/zein films did not significantly affect the contact angle [81]. However, the addition of basil essential oil (0.1–0.2%) to GG films increased the contact angle, potentially due to the hydrophobic coating effect of the oil on the film surface [80]. The inclusion of *Clitoria ternatea* anthocyanin in GG films increased the contact angle, and the addition of soy protein further enhanced this effect [73]. This observation is attributed to the disruption of the hydrogen bonding network within the GG matrix, altering the surficial properties. The incorporation of eggshell nanoparticles into GG films also led to a substantial increase in contact angle [48]. These nanoparticles act as fillers, hindering water penetration and increasing the hydrophobicity of the films.

These findings underscore the complex interplay between GG film composition and surface hydrophobicity. Future research should investigate the effects of adding different gelling bases to GG films along their contact angle. Additionally, a comprehensive understanding of the mechanisms underlying the observed changes in contact angle would facilitate the development of GG-based films with properties tailored to specific applications. For example, films with higher contact angles could be advantageous for applications requiring moisture resistance, while films with lower contact angles might be preferred for applications where water absorption is desirable.

### 3.3. Oxygen Permeability

The ability of packaging materials to restrict oxygen transfer is crucial for preserving food quality and extending shelf-life. Oxygen, a primary factor in food spoilage through processes like oxidation, can significantly degrade nutrients and flavor profiles in stored food products. Hence, designing packaging with excellent oxygen barrier properties is essential for maintaining the freshness and nutritional value of packaged foods. Various methods have been employed to assess the oxygen permeability of films, each providing insights into the effectiveness of the packaging material under different conditions. For instance, the oxidation of edible oils, measured by peroxide value, reflects the capability of the film to control oxygen transfer [81]. Other techniques include the use of deoxidizer absorption methods [38] and direct measurements with a film-package permeability tester [38], offering quantitative data on the amount of oxygen that can penetrate the films.

The composition and structural design of GG films significantly influence their oxygen barrier properties. For example, blackberry extract-integrated GG films exhibited an oxygen permeability of approximately 7.12 cm^3^·μm·m^−2^·day^−1^·kPa^−1^, which decreased to about 5.57 m^3^·μm·m^−2^·day^−1^·kPa^−1^ when configured into a bilayer film incorporating gelatin. The enhanced performance of the bilayer film is attributed to the formation of a perpendicular layer that obstructs oxygen passage more effectively [38]. Similarly, films made from GG combined with different starch types, such as cassava or maize, demonstrated varying degrees of oxygen permeability, with cassava starch films showing notably lower permeability than those made with maize starch [76]. This suggests that the choice of starch in the composite can be optimized based on the desired barrier properties.

The addition of functional compounds like rosemary essential oil and nisin has been shown to alter the oxygen permeability of GG films. For instance, the addition of 20% rosemary essential oil reduced the oxygen permeability of a GG/zein film from 44.4 to 39.4 mEq hydroperoxide/kg oil [81]. The incorporation of nisin (3912 IU/cm^2^ in films) into GG/pectin films increased the oxygen permeability from 0.95 × 10^−12^ to 3.44 × 10^−12^ g·m·m^−2^·s^−1^·Pa^−1^ [65]. These indicate that the specific interactions between the polymer matrix and the functional additives can significantly impact the oxygen permeability. Moreover, the construction of double-layer films can further enhance barrier properties. A double-layer film containing roselle anthocyanin and a nisin-incorporated cellulose/starch composite showed an ~47% reduction in oxygen permeability compared to its single-layer counterpart [67]. Additionally, films incorporating nano/micro fillers like *Broussonetia papyrifera* fruit anthocyanin and mica nanosheets exhibited a 90-fold reduction in oxygen permeability, underscoring the effectiveness of nano-fillers in creating a tortuous path that hinders gas molecule movement and enhances barrier function [55].

The diverse findings in oxygen permeability studies of GG films highlight the need for further research that systematically investigates the impact of different film compositions and functional additives. This research is crucial for developing GG-based films with oxygen barrier properties tailored to specific food packaging applications. A deeper understanding of the underlying mechanisms governing oxygen permeability in these films will enable the design of innovative packaging solutions that effectively extend the shelf-life and maintain the quality of various food products.

### 3.4. Optical Properties

**Figure 6:**
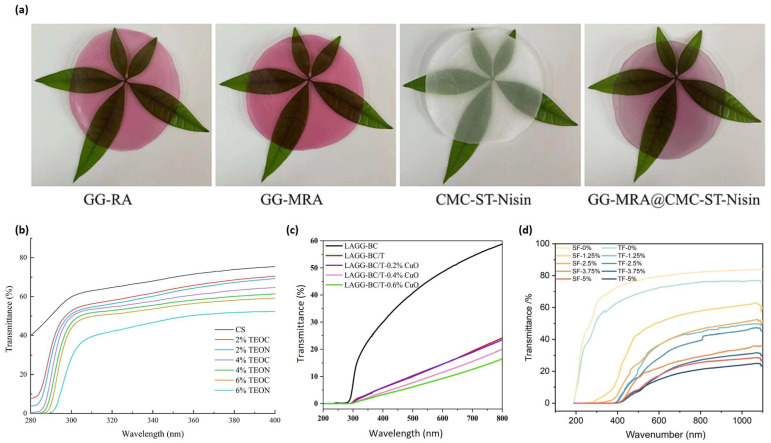
Optical properties of gellan gum-based composites. (**a**) Opacity of gellan gum composites with roselle anthocyanin and nisin [67]. (**b**) Gellan gum/chitosan composites containing thyme essential oils [58]. (**c**) Gellan gum/cellulose composites containing titanium oxide and copper oxide nanoparticles [31]. (**d**) Gellan gum/polyvinyl alcohol single-layer and triple-layer composites containing *Alhagi sparsifolia* flower extract [74]. (**a**–**c**) were reprinted with permission from Elsevier. (**d**) was reprinted under a CC-BY 4.0 license.

Solar ultraviolet (UV) radiation is categorized into three spectral bands—UVC (200–280 nm), UVB (280–315 nm), and UVA (315–400 nm)—as defined by the Global Solar UV Index [99]. The spectrum of visible light, on the other hand, spans from 400 to 800 nm [100]. The optical properties of GG-based films are crucial for food packaging applications, as they directly impact the preservation and presentation of food products. A delicate balance is required between UV protection and visible light transparency. While UV radiation can degrade food quality and cause nutrient loss, consumers often prefer packaging that allows them to visualize the product. Neat GG composites usually exhibit the highest light transmittance compared to films made with bioactive compounds (Figure 6).

Several studies have demonstrated the influence of various additives on the optical properties of GG films. For example, increasing the concentration of red cabbage anthocyanins from 0% to 40% in GG films significantly reduced transmittance at 535 nm from 91% to 22%, indicating a substantial decrease in visible light transmission [43]. Similarly, the incorporation of thyme essential oils increased the opacity of GG films [58]. Other plant-based compounds, like Caucasian whortleberry extract [9] and dragon fruit peel [94], also increased the light transmittance of GG-based composites.

Inorganic nanoparticles like TiO_2_, CuO, ZnO, and SiO_2_ have also been shown to affect the optical properties of GG films. GG/cellulose films containing 0.4% TiO_2_ and 0.6% CuO exhibited strong UV barrier properties, with transmittance below 5% in the 300–400 nm range [31]. However, this also led to a decrease in visible light transmission, with less than 20% transmittance in the 400–800 nm range. Similar trends were observed with ZnO nanoparticles, where increasing concentrations decreased light transmittance in GG composite films [11]. The addition of SiO_2_ nanoparticles to GG/cellulose films also resulted in reduced light transmission at 600 nm [89]. Furthermore, the incorporation of chitosan nanoparticles into GG films led to a dose-dependent decrease in transmittance across the 200–900 nm range [101]. Similarly, eggshell nanoparticles increased the opacity and light absorbance of GG films [48].

These findings underscore the importance of carefully selecting and optimizing the type and concentration of additives to achieve the desired optical properties in GG-based films. While incorporating UV-blocking agents is essential for food preservation, it is equally important to maintain sufficient visible light transparency to meet consumer preferences. Future research could focus on developing GG-based films that provide optimal UV protection while minimizing the impact on visible light transmission. This could involve exploring novel additives, optimizing film formulations, or incorporating multi-layer structures to achieve the desired optical properties.

### 3.5. Antioxidant and Antimicrobial Properties of GG-Based Composites

#### 3.5.1. Antioxidant Properties

The antioxidant activity of GG-based films is relatively understudied. Previous studies predominantly used the 2,2-diphenyl-1-(2,4,6-trinitrophenyl) hydrazyl (DPPH) method to measure the antioxidant capability of GG-based composites [30,31,37,78,85]. Neat GG composites typically exhibit weak antioxidant activity [31]. However, incorporating functional components can significantly enhance their antioxidant properties. For instance, the addition of TiO_2_/CuO nanoparticles increased the DPPH radical scavenging activity of GG composites from 2.80% to 23.37% [31]. In GG films containing cranberry extract and *Lactococcus lactis*, the antioxidant activity appeared to be primarily influenced by the cranberry extract’s concentration, with the addition of *Lactococcus lactis* having no significant effect [30]. The incorporation of konjac glucomannan into GG films containing gallic acid enhanced the DPPH radical scavenging activity from 90.78% to 93.55%, attributable to the formation of a dense network structure that increased loading capacity [37]. Similarly, the addition of dihydromyricetin to GG/konjac glucomannan films significantly improved DPPH radical-scavenging activity to approximately 90% [85]. GG films containing black rice anthocyanin/curcumin showed DPPH a radical scavenging activity of 85.64–97.06% [49]. The enhanced antioxidant activity of GG-based composites was also observed with the addition of rosemary essential oil [78,81] and mulberry extract [96].

These findings suggest that the antioxidant activity of GG-based films can be tailored through the incorporation of various functional compounds. However, further research is needed to fully understand the mechanisms involved and to optimize the film compositions for specific applications.

#### 3.5.2. Antimicrobial Properties

GG-based composites showed antimicrobial effects against diverse bacteria and fungi to different extents (Table 3). The bacterial strains used in previous studies include *Bacillus anthracis* [38], *Bacillus subtilis* [60], *Colletotrichum gloeosporioides* [36], *Cronobacter sakazakii* [89], *Escherichia coli* [102], *Fusarium* sp. [36], *Listeria monocytogenes* [65], *Pseudomonas aeruginosa* [35], *Pseudomonas fluorescens* [78], *Salmonella enteritidis* [56], *Salmonella* Typhimurium [78], and *Staphylococcus aureus* [41]. Antimicrobial testing of GG-based films was also conducted on mold, including *Alternaria alternata* [103], *Aspergillus niger* [13], *Botryotinia fuckeliana* [103], and *Botrytis cinerea* [104], and yeast, like *Candida* spp. [64,75,88]. Neat GG films generally showed no antimicrobial effects [34,35,36,37,78]. The incorporation of functional compounds significantly improves the antimicrobial effects of GG-based composites.

GG-based composites containing inorganic materials exhibit antimicrobial effects. The addition of 1–3% TiO_2_ nanoparticles to GG/k-carrageenan/xanthan gum showed no antimicrobial activity against *S. aureus*, while the film containing 5% TiO_2_ began to show antimicrobial effects (inhibition diameter: 15.5–16.5 mm) [34]. On the other hand, GG film with 1% TiO_2_ showed antimicrobial activity against *S. aureus*, *E. coli*, and *P.* aeruginosa, with inhibition zones of 10, 12, 11, and 10 mm, respectively [35]. This discrepancy should be verified by future studies. The GG/polyacrylamide films containing 1% ZnO nanoparticles showed no inhibitory effect against bacteria including *Bacillus cereus*, *Cronobacter sakazakii*, *E. coli*, *Salmonella enterica*, *Salmonella* Typhimurium, and *S. aureus*, while the growth of these strains was suppressed with the addition of 3% and 5% ZnO nanoparticles [11]. Among these strains, *S. aureus* was more susceptible to the composites than other strains, with inhibition zones of 32–36 mm [11]. These strains were also inhibited by the GG/cellulose film containing SiO_2_ nanoparticles, with a more profound effect on *S. aureus* (inhibition zone: 34 mm) [89]. GG films containing Ag nanoparticles exhibited antifungal effects against *C. albicans*, *C. glabrata*, *C. haemulonii*, *C. krusei*, and *C. lusitaniae* [88].

The antimicrobial effects of essential oils in the GG-based composites are strain-specific. GG/cellulose composites containing rosemary essential oil had antimicrobial effects against *E. coli*, *P. fluorescens*, *S. aureus*, and *Salmonella* Typhimurium [78]. Specifically, *S. aureus* (inhibition zone: 15.85 mm) showed the highest susceptibility to the composites, followed by *Salmonella* Typhimurium (inhibition zone: 15.41 mm), *E. coli* (inhibition zone: 14.11 mm), and *P. fluorescens* (inhibition zone: 12.61 mm) [78]. Another study found that *Botryotinia fuckeliana* was more sensitive than *Alternaria alternata* to the thyme essential oil-incorporated GG film [103]. The form of essential oil also affects its antimicrobial capabilities. For example, the GG/chitosan film incorporating 6% thyme essential oil nanoemulsion exhibited higher antimicrobial activity than that with 6% thyme essential oil coarse emulsion [58]. The reduced particle size characteristics of nanoemulsions potentially enhance the interaction of essential oil with cell membranes, which may facilitate the disruption of membrane permeability by the hydrophobic essential oil molecules. GG-based composites containing clove essential oil [64], myrtle essential oil [9], mustard essential oil [38], and dill essential oil [13] also showed antimicrobial effects against diverse strains.

Other functional compounds also showed antimicrobial effects in GG-based composites. Gram-negative bacteria (*E. coli*) were more susceptible to GG/konjac glucomannan films containing gallic acid than Gram-positive bacteria (*S. aureus*) [37]. Conversely, GG/konjac glucomannan films containing dihydromyricetin showed more profound effects against Gram-positive bacteria than against the Gram-negative bacteria tested [85]. A GG film containing norfloxacin (0.01–1%) inhibited the growth of *E. coli* (inhibition zone: 5.3–13.0 mm) and *S. aureus* (inhibition zone: 5.0–13.3 mm) [105]. Probiotic strains also showed antimicrobial effects in the GG composites. For example, the incorporation of *Lactococcus lactis* (1–2%) into the GG film inhibited the growth of *Listeria monocytogenes* in a concentration-dependent manner (inhibition zone increased from 7.0 to 14.9 mm) [30]. The possible explanation for this is that the strain may produce bacteriocins that can inactive pathogens. GG-based composites containing lactoferrin also showed antimicrobial effects [70,71].

While the antimicrobial properties of GG-based composites against bacteria and fungi have been extensively studied, there is a notable gap in the research regarding their effects on viruses. Given the global emphasis on infection control, particularly in light of recent pandemics, investigating the antiviral capabilities of GG-based materials could significantly broaden their applications in healthcare, food safety, and public sanitation. In addition, the plant extracts from biowastes and agro-industrial wastes may be possible additives that can be used in the GG-based packaging materials as active agents, contributing to the valorization of these wastes or byproducts.

### 3.6. Release Properties of GG-Based Composites

The release of functional compounds from GG-based polymer films can be described as a two-stage process. Initially, liquid molecules infiltrate the polymer matrix, causing swelling and the weakening of the polymer network. Subsequently, the functional molecules migrate from within the film to the surrounding solution until a state of thermodynamic equilibrium is reached. Studies have shown varying release patterns for different compounds.

The release of rosemary essential oil from GG/zein films was studied by immersing the active films into hydrophilic (10% ethanol) and fatty (95% ethanol) food stimulants [81]. The release rate into fatty food stimulants at both 4 °C and 24 °C increased from 0 to 24 h and then plateaued. The equilibrium release rate was higher at 24 °C compared to 4 °C. In hydrophilic food stimulants, the release rate reached a maximum after 3 h and remained stable for 72 h, with higher releases at 24 °C compared to 4 °C. The higher release rate in fatty food stimulants compared to hydrophilic ones is attributed to the hydrophobic nature of rosemary essential oil [81]. GG/polyvinyl alcohol films incorporating *Alhagi sparsifolia* flower extracts showed different release rates in terms of antioxidative capacities in various food-simulating liquids [74]. After 96 h, the release order was 10% ethanol > 3% acetic acid > pure water > 95% ethanol. Single-layer films had a higher initial release rate, while triple-layer films showed a more sustained release over time, indicating the potential for controlled release applications [74].

Nisin diffusion from GG/pectin films was modeled using the Crank equation, with diffusion coefficients (D) of 7.36 × 10^−14^ m^2^/s at 30 °C and 5.22 × 10^−14^ m^2^/s at 5 °C [65]. Higher temperatures increased the D-value, with over 75% of nisin released within the first 72 h, before reaching equilibrium [65]. GG/starch/polyester-based bilayer films showed the complete recovery of initially incorporated ferulic acid after about 6 h of contact time [10]. The release pattern followed Peleg’s model, indicating a predictable and consistent release behavior [10].

The release of *Clitoria ternatea* anthocyanins from GG/soy protein films was slower at pH 7.4 compared to pH 1.2 [73]. At alkaline pH values, covalent interactions between the quinonoidal base of anthocyanins and the sulfhydryl and amino groups of soy protein resulted in stronger binding, leading to slower release despite higher swelling ratios [73]. GG/konjac glucomannan composite films released 42–75% of the total incorporated gallic acid in distilled water at 25 °C. This suggests that some gallic acid remains physically trapped within the network structure of the composite [37]. The release of purple sweet potato anthocyanins from GG composite films was faster at pH 7.4 and pH 6.0 compared to pH 2.0 [97]. This is due to the carboxylic acid groups in GG forming hydrogen bonds in acidic conditions, causing hydrogel shrinkage. At higher pH levels, these groups convert into carboxylate ions, resulting in swelling and faster release [97].

These studies demonstrate that GG-based composites can control the release of various functional compounds, making them suitable for applications in food packaging and preservation. The release properties can be tailored by modifying the composition and environmental conditions, such as temperature and pH. This adaptability allows for the development of packaging materials that can provide the sustained release of active agents, enhancing the shelf-life and safety of food products. Future research should focus on exploring their release behaviors under different environmental conditions and with a wider range of functional compounds. Understanding the interactions between GG and these compounds will help in designing more effective and versatile packaging solutions.

### 3.7. Degradability of GG-Based Composites

**Figure 7:**
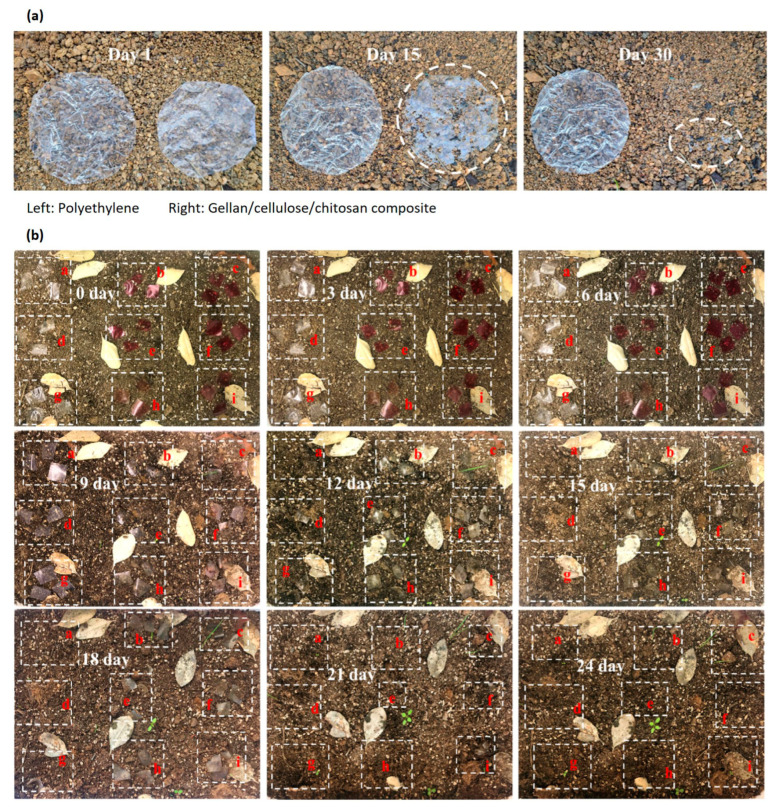
Degradability of gellan-based composites. (**a**) Degradation of gellan gum/cellulose/chitosan composites [32]. Reprinted with permission from American Chemical Society. (**b**) Degradation of gellan gum films containing cranberry extract and *Lactococcus lactis* [30]. Reprinted with permission from Elsevier.

Few studies have explored the degradability of GG-based composites, yet those that exist provide promising insights into their environmental impact and potential for use in sustainable applications (Figure 7). Pure GG films, being hydrophilic, were completely degraded in soil within 15 days [30]. The degradation process was slowed down when cranberry extract and *Lactococcus lactis* were incorporated into the films. This slowdown was attributed to the antibacterial and antioxidant agents present in the films, which inhibited microbial activity in the soil, thereby reducing the rate of degradation [30]. The aerobic biodegradability of GG/cellulose nanocrystals films was assessed through the accumulation of carbon dioxide using the UNE-EN ISO 17556 standard methodology [106]. Over a 90-day period, the accumulation of carbon dioxide increased in a time-dependent manner, indicating the sustained degradation of the films [106]. This suggests that GG/cellulose composites maintain their degradability over extended periods of time under aerobic conditions. GG/cellulose/chitosan multi-layer films also demonstrated effective biodegradation in soil with a moisture content of about 13% at room temperature [32]. Noticeable breaks in the surface of the film occurred after 15 days, and complete degradation was observed within 30 days [32]. This indicates that even multi-layered composites, which might be expected to have more complex degradation behaviors, can break down efficiently in natural environments.

These studies suggest that GG-based films are biodegradable, although the rate and extent of degradation can be influenced by the composition of the film and the environmental conditions. Future research should explore degradability under diverse conditions, such as sand and water simulations, and investigate the effects of various functional compounds on the degradation process. This information is critical for developing sustainable and environmentally friendly GG-based materials for use in food packaging and other applications.

## 4. Active Packaging of GG-Based Packaging Materials

### 4.1. Active Packaging for Fruits and Vegetables

**Figure 8:**
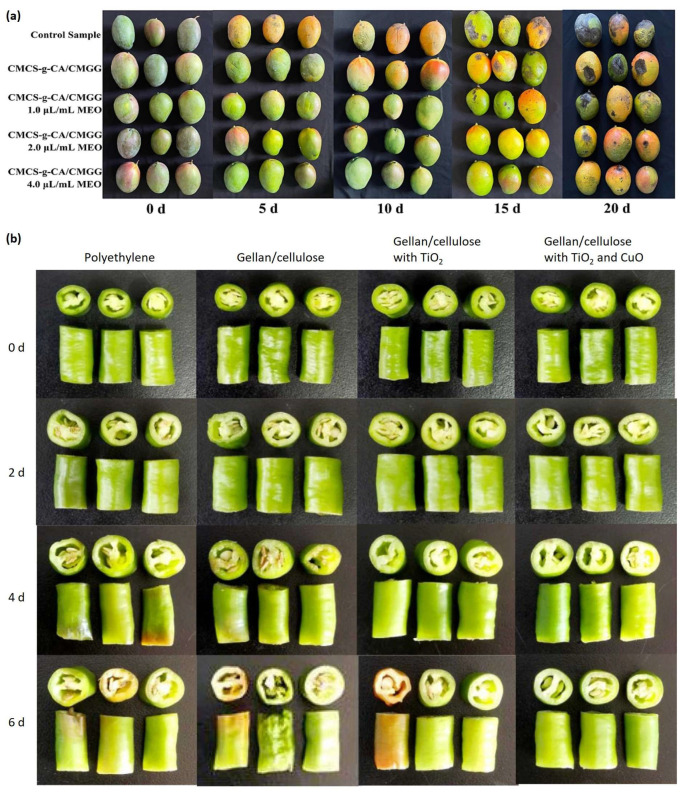
Preservation of fruits and vegetables by gellan gum-based packaging materials. (**a**) Mangos coated with gellan gum/chitosan composites enriched with mustard essential oil [38]. (**b**) Fresh-cut peppers wrapped in gellan gum/cellulose films containing titanium oxide and copper oxide nanoparticles [31]. Both figures were reprinted with permission from Elsevier.

GG showed protective effects on fruits and vegetables (Table 4) (Figure 8). The fruits and vegetables used in previous studies include *Agaricus bisporus* mushroom [91,107], apple [82,104], apricot [8], banana [59], blueberry [54,63,84], jackfruit [108], litchi [109], mandarin [39], mango [38], melon [110], persimmon fruit [82,104], strawberry [70], and tomato [82]. The following subsections discuss the effects of GG films/coatings on the quality attributes of these fruits and vegetables.

#### 4.1.1. Firmness

The application of GG-based coatings has shown significant promise in enhancing the firmness of various fruits and vegetables during storage. Firmness is an important quality attribute, often associated with the freshness and textural appeal of produce. For instance, GG/chitosan composite films enriched with mustard essential oil were effective in maintaining the hardness of mangoes at 5.66 N after 20 days of storage [38]. Similarly, the firmness of GG-coated bananas was recorded at 60.41 N after a 5-day storage period, marking a notable increase compared to the 36.75 N of uncoated bananas [59]. This demonstrates the coating’s ability to significantly reduce softening over time. In a study on mushrooms coated with a GG film incorporating cellulose nanocrystals, the final firmness was measured at 1.7 N on day 20, compared to 0.5 N for the uncoated sample [91]. Apricots treated with GG exhibited a firmness of 1.98 N at the end of the storage period, significantly higher than the 1.46 N of the control apricots [8]. Firmness retention was also observed in apple slices, where uncoated slices had a firmness of 65.5 N/cm^2^ after 12 days, while those coated with vanillin-incorporated GG showed a firmness of 70.2 N/cm^2^ [83]. Fresh-cut melon maintained a firmness of 2.06 N after 28 days at 4 °C, compared to 1.71 N for the uncoated samples [110]. Similar results were observed for mandarin [39]. The improved firmness in coated fruits and vegetables can be attributed to the reduction in cell wall polysaccharide-degrading enzymes. GG/konjac glucomannan coatings, for example, have been shown to reduce the activity of enzymes such as cellulase, pectin lyase, pectinesterase, polygalacturonase, xyloglucan endotransglycosylase, α-arabinofuranosidase, α-mannosidase, β-galactosidase, β-glucoamylase, and β-xylanase in blueberries [63]. This reduction in enzymatic activity helps maintain the structural integrity of cell walls, thereby preserving firmness during storage.

#### 4.1.2. Weight Loss

GG-based coatings have been shown to effectively reduce weight loss in various fruits and vegetables during storage. For example, coating apricots with GG resulted in a significant reduction in weight loss, falling to 4.7% after 15 days of storage compared to 26.85% for the control samples. This weight loss rate was even lower than those for alginate (5.8%) and chitosan (6.84%) coatings [8]. In another study, the application of TiO_2_/CuO-incorporated GG coating to fresh-cut peppers resulted in a weight loss rate of 17.31%, which was significantly lower than the 23.22% observed for samples wrapped in polyethylene [31]. Similarly, mandarin oranges coated with oregano essential oil-incorporated GG exhibited a weight loss rate of 0.46–0.49% after 30 days of storage, compared to 0.58% for the uncoated samples [39]. Strawberries also benefited from GG-based coatings, with coated samples showing about 60% weight loss after 144 h of storage, whereas uncoated samples experienced approximately 90% weight loss [70]. Similar positive effects on weight loss were observed in mangoes [38]. These results suggest that GG-based coatings can significantly reduce the rate of weight loss in various types of produce, thereby preserving their quality and extending their shelf-life. The effectiveness of these coatings can be attributed to their ability to form a semi-permeable barrier that reduces moisture loss.

#### 4.1.3. Respiration

GG-based coatings can impact the respiration rates of various fruits and vegetables, thereby enhancing their storage stability. The respiration rate, indicated by the production of CO_2_ and the consumption of O_2_, is a critical factor affecting the shelf-life and quality of fresh produce.

In bananas, the production rate of CO_2_ increased linearly up to 40 h before declining, becoming negligible after 48 h. In contrast, coated bananas exhibited a lower initial rate of CO_2_ production, which gradually increased up to 140 h [59]. The coating reduced the exposure to O_2_ and slowed the respiration rate, especially during the initial days of storage, thereby enhancing the storage stability of the bananas [59]. Mushrooms showed a significant reduction in oxygen consumption when coated with GG films incorporating cellulose nanoparticles [107]. The uncoated mushrooms had an oxygen consumption rate of 400 mL O_2_/kg day on day 3 and 270 mL O_2_/kg day on day 10. In contrast, mushrooms with a GG coating containing 20% cellulose nanoparticle showed oxygen consumption rates of 50 mL O_2_/kg day and 20 mL O_2_/kg day on days 3 and 10, respectively. The CO_2_ levels in coated mushrooms were also maintained at lower levels (75 and 40 mL/kg day on days 3 and 10) compared to the control mushrooms (220 and 170 mL/kg day) [107]. Similar beneficial effects on respiration rates were observed in jackfruit coated with GG during a 9-day storage period [108]. However, the effect of coatings can vary depending on the type of fruit. For instance, coatings tended to increase the O_2_ consumption and CO_2_ production rates in apples, and they had no significant effect on the respiration rates of persimmons [104]. The reason for the discrepancy with results from previous studies remains to be studied. These findings suggest that GG-based coatings can effectively modulate the respiration rates of certain fruits and vegetables, thereby enhancing their storage stability and extending their shelf-life.

#### 4.1.4. Phenolic Compounds

Total phenolic content (TPC) is an important indicator of the nutritional quality and antioxidant capacity of fruits. The application of GG-based coatings has been found to effectively preserve phenolic compounds in various fruits during storage, reducing the degradation caused by oxidative processes and enzymatic activities. In a study on mandarins, the control samples exhibited a significant decline in TPC, with a reduction of 55.68% from harvest to the last storage day (24 day). In contrast, mandarins coated with oregano essential oil-incorporated GG coatings showed a much lower rate of TPC reduction, ranging from 20.51% to 24.52% [39]. Similarly, GG-coated apple slices retained higher TPC after 12 days of storage, with a recorded value of 94.3 mg GAE/100 g compared to the value of 81.5 mg GAE/100 g attained in uncoated slices [83]. In the case of jackfruit bulbs, the application of GG coatings showed a biphasic response [108]. Initially, coated samples showed lower total soluble phenolic contents than uncoated ones, potentially due to the delayed ripening and inhibition of polyphenol oxidase activity caused by GG and 1-methylcyclopropene [108]. However, in later storage stages, the coated samples exhibited higher TPC levels than uncoated ones. This suggests that while GG coatings might initially delay phenolic compound accumulation, they could ultimately contribute to their preservation in the long run [108].

The ability of GG-based coatings to preserve phenolic compounds can be largely attributed to their semi-permeable nature, which controls gas exchange and reduces the oxidative stress on the fruit tissues. This not only helps in maintaining the nutritional quality of the fruit but also enhances its shelf-life by slowing down the degradation processes. Further research is needed to elucidate the precise mechanisms underlying the interaction between GG coatings and phenolic compounds in different fruits. Understanding these mechanisms could lead to the development of tailored coating formulations to optimize the preservation of TPC and other quality attributes in fresh produce.

#### 4.1.5. Ascorbic Acid

The preservation of ascorbic acid (vitamin C) is important for maintaining the nutritional quality and antioxidant properties of fruits during storage. GG-based coatings have demonstrated effectiveness in reducing the loss of ascorbic acid in various fruits, thereby maintaining their nutritional value.

In a study on mandarins, the ascorbic acid content in the control samples decreased by 2.08% by day 14 at 6 °C plus 7 days at 15 °C compared to day 0. In contrast, the coated samples exhibited an increase of 9.12–11.56% compared to day 0 [39]. This indicated that the GG-based coating not only preserved but also enhanced the ascorbic acid content, possibly due to the reduction in oxidative stress and enzymatic degradation. In mangoes, the ascorbic acid content dropped from 86.99 mg/100 g to 38.02 mg/100 g in mustard essential oil-incorporated GG/chitosan coated samples after 10 days of storage, while the uncoated samples had a larger decrease to 49.64 mg/100 g [38]. The lower reduction in coated samples underscores the efficacy of GG coatings in maintaining ascorbic acid levels. GG coating was more effective in reducing ascorbic acid loss (20.4%) in apricots compared to chitosan (21.6%) and alginate (25.0%) coatings after 15 days of storage [8]. This highlights the superior protective effect of GG coatings in preserving ascorbic acid. GG coatings delayed the peak of ascorbic acid content in jackfruit, with the maximum level observed on day 8 for coated samples compared to day 4 for uncoated samples [108]. This indicates that GG coatings could influence the ripening process and delay vitamin C degradation in jackfruit. However, in the case of melon stored at 4 °C for 28 days, the ascorbic acid content in GG-coated samples showed similarity to that of uncoated samples [110]. This suggests that the effectiveness of GG coatings may vary depending on the type of fruit and storage conditions.

In conclusion, the effect of GG coatings on ascorbic acid content in fruits during storage appears to be context-dependent, varying with fruit type, coating composition, and storage conditions. While GG coatings have shown promise in preserving or even enhancing vitamin C levels in some fruits, their effectiveness may be limited in others. Additionally, GG coatings can influence the ripening process, which can indirectly affect vitamin C levels. Further research is needed to understand the underlying mechanisms by which GG coatings interact with ascorbic acid in different fruits. This knowledge could facilitate the development of targeted coating strategies to optimize vitamin C preservation and overall fruit quality during storage.

#### 4.1.6. Microbial Growth

The antimicrobial efficacy of GG-based coatings in food preservation varies depending on the target microorganisms, coating formulation, and food matrix. In strawberries, the use of carboxymethylcellulose-incorporated lactoferrin/chitosan/GG composite coatings significantly reduced bacterial populations [70]. After 144 h of storage, the bacterial count in the treated strawberries was 3.51 log CFU/mL, which was lower than that in the control samples, at 4.2 log CFU/mL [70]. Similar antibacterial effects were observed in coatings incorporating carboxymethylcellulose into lactoferrin/GG composites [71].

Fresh-cut strawberries treated with GG-based coatings incorporating geraniol showed a significant antimicrobial effect, reducing the counts of mesophilic bacteria, yeast and molds, and psychrophilic bacteria compared to control samples and those treated with neat coatings or coatings with pomegranate extract [86]. Similarly, GG coatings with geraniol exhibited a significant bacteriostatic effect on fresh-cut apples from day 8 of storage, with bacterial counts 1.4–1.7 log lower than those of neat GG-coated apples [83]. These studies support the use of geraniol as a natural antimicrobial agent in combination with GG coatings for the preservation of fresh produce.

Despite the generally positive results, there are instances where GG-based coatings do not significantly reduce microbial growth. For example, GG/starch coatings did not significantly reduce the disease incidence in apples inoculated with Botrytis cinerea, as compared to non-coated control apples, after 7 or 12 days of storage at 20 °C [104]. This suggests that the efficacy of GG-based coatings can vary depending on the type of pathogen and the specific formulation of the coating.

The antimicrobial effectiveness of GG-based coatings depends on multiple factors, including the type of microorganism, the food matrix, and the specific formulation of the coating. While GG alone may offer some antimicrobial activity, combining it with other natural antimicrobials like essential oils or plant extracts can significantly enhance its efficacy. Future research should focus on optimizing the composition and application of GG-based coatings for different food products and storage conditions.

#### 4.1.7. Browning Progress

GG-based coatings, especially when enriched with bioactive compounds, have shown effectiveness in reducing the browning of fresh-cut fruits and vegetables. For instance, untreated fresh-cut potatoes and apples displayed obvious surface browning and severe shrinkage due to water loss after six days of storage [30]. In contrast, pure GG treatment had minimal impact in terms of improving the quality of these samples. However, when GG coatings were incorporated with cranberry extract and Lactococcus lactis, the visible browning degree in fresh-cut potatoes and apples was significantly lower compared to untreated samples [30]. This indicates that the addition of these bioactive components can enhance the anti-browning properties of GG coatings. The hue angle (*h*°), which is a measure of color, further supports these findings. Uncoated fresh-cut apple cubes stored at 5 °C showed a decrease in hue angle to 77.51 by day four, indicating significant darkening retention [83]. Conversely, the hue angle of apple cubes coated with GG and vanillin only decreased to 80.04, suggesting a slower browning process and better color retention [83]. Browning in fruits and vegetables is a significant quality issue, primarily driven by the activity of polyphenol oxidase (PPO) and peroxidase (POD) [112]. In apricots, the enzymatic activities associated with browning were markedly reduced by GG coatings. After 10 days of storage, the PPO activity in GG-coated apricots was one-tenth of that in uncoated samples, and POD activity was reduced to one-fifth of that in the uncoated samples [8]. This substantial reduction in enzymatic activity highlights the efficacy of GG-based coatings in controlling browning. These findings suggest that GG-based coatings can be a valuable tool in the fresh-cut produce industry, extending shelf-life and enhancing product appeal.

### 4.2. Active Packaging for Starch-Based Food Products

**Figure 9:**
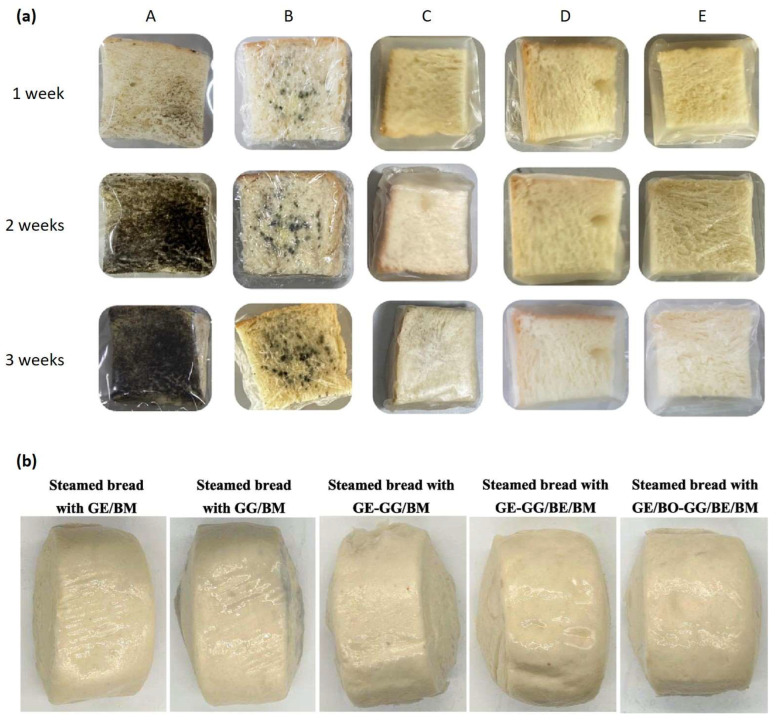
Preservation of starch-based food products using gellan gum-based packaging materials. (**a**) Bread, protected with gellan/cellulose, that contains *Anethum graveolens* essential oil stored at room temperature. A, polypropylene bags; B, polypropylene wraps; C, D, E, gellan/cellulose films incorporating 0%, 2%, and 4% of essential oil, respectively [13]. (**b**) Chinese steamed bread coated with different gellan gum-based composites. BE, blackberry extract; BM, *Bifidobacterium longum*; BO, baobab seed oil; GE, gelatin; GG, gellan gum [40]. Both figures were reprinted with permission from Elsevier.

In addition to fruits and vegetables, GG-based coatings have demonstrated promising results in extending the shelf-life and maintaining quality of starch-based food products, such as tortilla [64], bread [13], and rice cake [111], and *mantou* (Chinese steamed bread) [40] (Figure 9). A study showed that tortillas coated with clove oil and natamycin-incorporated GG/pectin composites exhibited significantly lower microbial counts compared to uncoated controls [64]. The total aerobic count was reduced to 5.5 log CFU/g and the count for yeasts and molds fell to 4.8 log CFU/g, representing reductions of 2 and 3 logs, respectively, compared to the controls [64]. GG films incorporating *Anethum graveolens* essential oil effectively inhibited the growth of *Aspergillus niger* on bread during 3 weeks of storage [13]. These findings underscore the potential of GG-based coatings, combined with essential oils and other antimicrobial agents, to extend the shelf-life and safety of food products by reducing microbial growth. GG-based coatings significantly reduced moisture loss in rice cakes (1.08%) compared to uncoated samples (3.36%) after four days of storage at 25 °C and 45% relative humidity [111]. The coated rice cakes also exhibited a lower hardness (144.45 N) than the uncoated ones (225.21 N), suggesting that the coating might influence texture. Furthermore, the coating decreased the retrogradation rate by 9.73%, indicating the potential to delay starch retrogradation and maintain quality during storage [111]. These studies highlight the potential of GG-based coatings as a natural and effective strategy to extend shelf-life, maintain quality, and reduce food waste for staple foods. A study examined the survival of probiotics on coated *mantou* stored at 4 °C for 7 days [40]. The results showed that the viable cell numbers of *B. longum* in *mantou* with a single-layer GG film exhibited a general downward trend during the storage period. In contrast, the *mantou* with a bilayer composite coating maintained stable in terms of viable cell numbers of *B. longum*, with satisfactory levels recorded after 7 days (approximately 8.12–8.19 log CFU/g) [40]. However, further research is needed to understand how the probiotic-enriched coating affects the sensory quality of *mantou*.

### 4.3. Active Packaging for Animal-Derived Food Products

During meat spoilage, thiobarbituric acid reactive substances (TBARS) and malondialdehyde (MDA) are formed as byproducts of lipid oxidation, indicating rancidity. Total volatile basic nitrogen (TVB-N) results from protein degradation, producing ammonia and amines. Peroxide value (PV) measures the initial stages of lipid oxidation, where peroxides and hydroperoxides form. These compounds signal spoilage through oxidative and microbial degradation processes, leading to off-flavors, odors, and reduced meat quality. Monitoring these indicators helps to assess meat freshness and shelf-life. The shelf-life of animal-derived food products can be extended using GG-based composites (Table 5).

#### 4.3.1. Active Packaging for Pork

**Figure 10:**
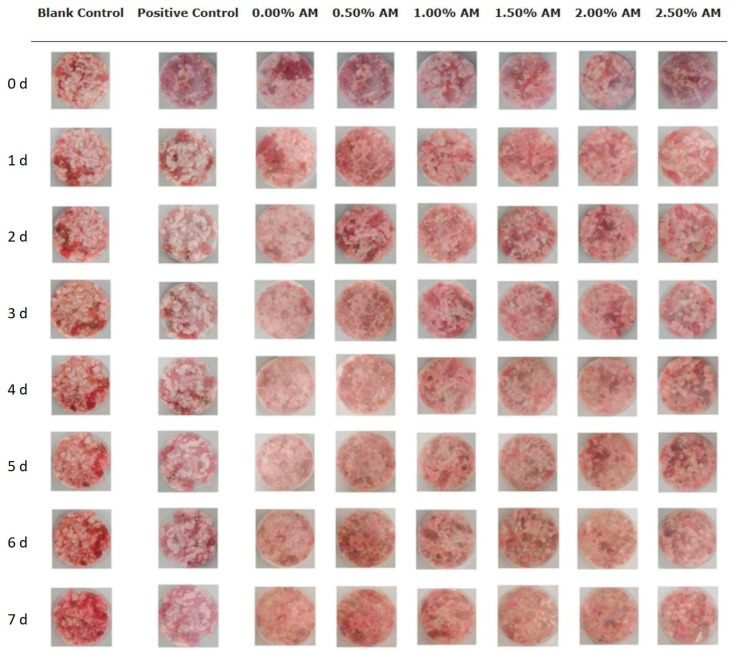
Minced pork protected with *Aronia melanocarpa* extract-incorporated gellan gum/pea protein/chitosan bilayer films [72]. This was reprinted under a CC-BY 4.0 license.

A previous study found that the greatest fresh pork weight losses throughout the cold storage period were observed in samples packaged in single-layer films, which exhibited significantly higher water vapor transmission rates compared to bilayer films with phenolic acids) [26]. The addition of phenolic compounds such as ferulic, *p*-coumaric, and protocatechuic acid to GG-based composites resulted in a higher weight loss (~11.5%) in pork stored for 16 days compared to samples with neat GG-based films (~5–10%) [26]. Furthermore, the addition of protocatechuic acid to GG bilayer films resulted in lower TBARS (0.68 mg MDA/kg) and peroxide values (3.1 meq O_2_/kg) than films containing ferulic acid (0.80 mg MDA/kg for TBARs and 4.1 meq O_2_/kg for peroxide value) and *p*-coumaric acid (0.75 mg MDA/kg for TBARs and 4.0 meq O_2_/kg for peroxide value) [26]. In another study, incorporating 1.50% *Aronia melanocarpa* extract into GG composite films substantially reduced weight loss in fresh pork (~20–27%) compared to the control (37.72%) after 7 days of storage (Figure 10) [72]. This reduction aligns with the lower water vapor transmission coefficient of the composite film, indicating its effectiveness in moisture retention and quality preservation. Among the coatings tested, the composite film made with 1.50% *Aronia melanocarpa* extract exhibited the best inhibitory effect on TVN-B in pork, significantly reducing TVN-B content by 66.13% by the 7th day of storage [72].

#### 4.3.2. Active Packaging for Aquatic Foods

GG-based edible films and coatings have demonstrated promising potential in reducing lipid oxidation and preserving quality in various aquatic food products. Incorporating *Alhagi sparsifolia* flower extract into GG-based films significantly reduced lipid oxidation in shrimp, as evidenced by the lower peroxide values and TBARS compared to films without the extracts [74]. Notably, triple-layer films exhibited higher efficacy in retarding lipid oxidation than single-layer films [74]. Similarly, GG-based composites incorporating bioactive compounds such as anthocyanin, curcumin, and ZnO showed potential in extending the shelf-life of shrimp [41]. These composite coatings effectively extended the shelf-life of shrimp stored at 4 °C by one day. This was determined based on TVB-N and TBARS analyses [41]. Furthermore, the application of GG-treated coatings on snakehead fish demonstrated notable improvements in storage stability. The treated fish exhibited significantly lower thawing loss and higher pH values compared to both control and polyethylene-wrapped samples. Additionally, the TVB-N content was lower in GG-treated samples, indicating reduced protein degradation and spoilage [62]. These studies demonstrate the effectiveness of GG-based films and coatings in reducing lipid oxidation, preserving quality, and extending the shelf-life of various seafood products. The incorporation of natural extracts and bioactive compounds further enhances the functionality of these coatings, offering a promising approach for achieving sustainable and safe fishery food preservation.

#### 4.3.3. Active Packaging for Poultry

The use of GG composites containing anthocyanin and nisin significantly enhances the freshness and reduces spoilage in chicken breasts during storage [67]. On the eighth day of storage, the TVB-N content, an indicator of protein degradation, was markedly lower in the GG-treated samples (22.16 mg/100 g) compared to the control samples (30.45 mg/100 g) [67]. Similarly, the pH values were also lower in the treated samples (6.53) than in the control group (6.74), indicating slower spoilage. By the eighth day, the control chicken breasts were spoiled, while the treated samples remained in a sub-fresh state, though both were spoiled by the tenth day [67]. This illustrates the effectiveness of GG composites with bioactive compounds in delaying spoilage, albeit not indefinitely.

Similarly, in turkey breast meat, the use of active film (GG-based films incorporating Caucasian whortleberry extract and myrtle essential oil) significantly reduced deterioration compared to neat GG and polyethylene-wrapped samples over 15 days of storage [9]. Lower TBARS (1.95 mg MDA/kg) and peroxide values (1.20 meq peroxide/1000 g lipid) were observed in samples wrapped with the active film, indicating its effectiveness in retarding oxidative rancidity [9]. Moreover, the active film also reduced TVB-N values (21.4 mg N/100 g) compared to the control groups (39.8 and 39.2 mg N/100 g for polyethylene and neat GG, respectively), suggesting its ability to inhibit protein and amine degradation by bacterial spoilage [9].

#### 4.3.4. Active Packaging for Other Animal-Derived Food Products

The use of basil essential oil-integrated GG composite coatings on eggs significantly enhances their preservation during storage [80]. After 42 days, the weight loss of eggs coated with the composite was a mere 0.40%, compared to a substantial 5.64% in eggs coated with neat GG. This coating also effectively inhibited the reduction in Haugh unit and yolk index values, parameters indicative of egg freshness and quality, and mitigated the rise in pH. Additionally, the total aerobic plate count was considerably lower in the basil essential oil-integrated GG-coated eggs (4.45 log CFU/g) compared to uncoated samples (7.55 log CFU/g), demonstrating its antimicrobial efficacy [80].

In the context of cheese preservation, GG coatings have also shown promising results [29,42]. A GG coating was more effective in reducing the weight loss (~24%) of cheese during a 12-day storage period compared to the cheese samples treated with other polysaccharides such as κ-carrageenan, xanthan gum, and methylcellulose (~25–26%) [29]. Furthermore, while neat GG/pectin films partially inhibited the effects of harmful bacterial strains, like *Escherichia coli*, *Staphylococcus aureus*, and *Listeria monocytogenes*, on Mexican cheese, the incorporation of supernatants from *Streptococcus infantarius* fermentations and EDTA into these films resulted in the complete inhibition of these bacteria [42]. This highlights the potential of GG-based films, especially when enhanced with bioactive components, to significantly improve the microbiological safety and shelf-life of dairy products.

## 5. Intelligent Packaging of GG-Based Colorimetric Indicators

### 5.1. Mechanism of Colorimetric Films

The color change of the colorimetric films in intelligent packaging is primarily triggered by the interaction between the pH-sensitive components in the film and the volatile compounds released during food spoilage. In the case of meat products, the decomposition of proteins by bacteria and enzymes generates volatile basic gases like ammonia, trimethylamine, and dimethylamine, causing a shift towards alkaline pH values [93,113]. Conversely, in the case of milk, the spoilage process is characterized by acidification, resulting in a decrease in pH [44]. Colorimetric indicators embedded in the film respond to this acidic environment, signaling the deterioration of milk quality. For fruits and vegetables, respiration and microbial activity generate CO_2_, an acidic gas [53]. This leads to an acidified environment in the packaging, triggering color changes in pH-sensitive indicators incorporated into the film. Thus, the intelligent packaging of GG-based composites leverages the pH-responsive nature of various compounds, such as anthocyanins and other pH-sensitive dyes, to provide a visual indication of the freshness and quality of different food products. By monitoring the color changes in the film, consumers and retailers can readily assess the condition of the packaged food, contributing to improved food safety and reduced waste.

### 5.2. Colorimetric Properties of GG-Based Composites

**Figure 11:**
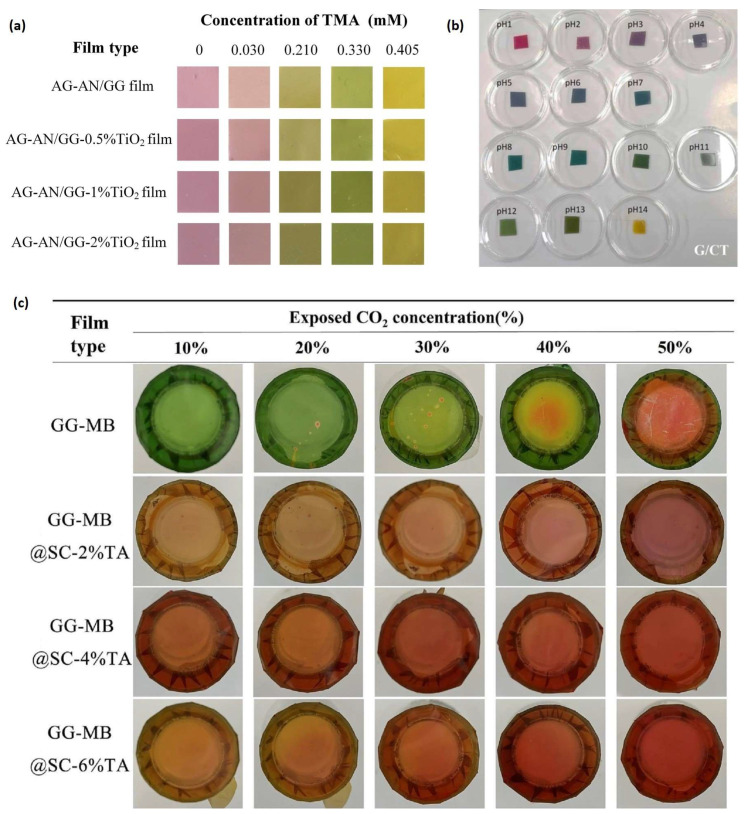
Colorimetric changes of different gellan gum-based indicators. (**a**) Rose anthocyanin-containing films responding to trimethylamine. AG, alginate; AN, anthocyanin; GG, gellan gum; TMA, trimethylamine [51]. (**b**) *Clitoria ternatea* anthocyanin-containing films responding to pH change [73]. (**c**) Methyl red/bromothymol blue-containing films responding to CO_2_ change. GG, gellan gum; MB, methyl red/bromothymol blue; SC, sodium carboxymethyl cellulose; TA, tannic acid [53]. All figures were reprinted with permission from Elsevier.

The pH-dependent color changes of GG-based indicators, made with anthocyanin pigments extracted from different plant sources, exhibit similar patterns, with distinct colors seen at various pH levels (Figure 11). For example, the color of GG film with *Clitoria ternatea* anthocyanins shifted from red at low pH values to violet, blue, blue-green, green, and brownish-yellow at increasing pH values [73]. Similarly, the film with red radish anthocyanins transitions from deep orange-red to light carmine, purple, yellow-green, and finally yellow as pH increases [44]. Purple sweet potato anthocyanins follow a comparable pattern, changing from red to violet or purple, blue, green, and yellow with increasing pH [97]. The color shifts of these flavylium-based pigments are attributed to the transformation of anthocyanin chemical structures, primarily from the flavylium cation (red) at low pH values to quinoidal bases (blue) and chalcones (yellow) at higher pH values. Carbon dioxide-sensitive films were developed [53]. The methyl red/bromothymol blue-incorporated films changed their color from brownish-yellow to purple-red in response to the varying concentrations of CO_2_, depending on the ingredients used (Figure 11c) [53].

The color stability of anthocyanin-incorporated films has been studied. The color stability of these composites is largely dependent on the temperature during storage. For example, the color change (Δ*E*) of *Broussonetia papyrifera* fruit anthocyanin-integrated GG/konjac glucomannan/carrageenan composites stored at 25 °C for 14 days was higher than that of the composites stored at 4 °C [55]. Similarly, the color of GG composites containing red cabbage anthocyanins was more stable at 4 °C than at 25 °C during 50-day storage [43]. Similar results for the negative effects of a higher storage temperature on the color stability of GG-based composites were also observed for studies involving mulberry anthocyanins [96], black rice anthocyanin [41], and red radish anthocyanins [44]. The degradation of anthocyanin structures at elevated temperatures is likely responsible for this phenomenon. In addition, illumination also accelerates the degradation of anthocyanins in the GG composites. For example, the retention rate of anthocyanins in a dark room for 10 h was above 92%, while it decreased to 51.76% and 76.21% for the samples exposed to UV light and natural light, respectively [94]. This suggests that light-induced photodegradation plays a significant role in the color instability of anthocyanin-containing composites.

### 5.3. Applications of GG-Based Colorimetric Indicators

#### 5.3.1. Intelligent Packaging for Aquatic Food Products

**Figure 12:**
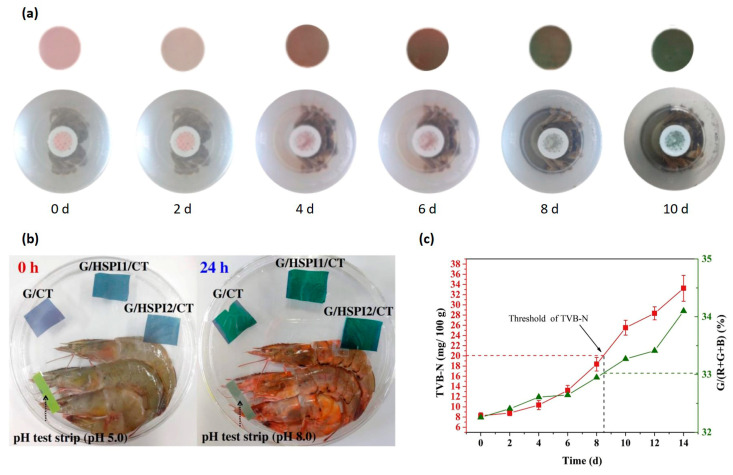
Freshness monitoring for aquatic food products. (**a**) Mulberry anthocyanin-containing film responding to spoilage of Chinese mitten crab [96]. (**b**) *Clitoria ternatea* anthocyanin-containing films responding to spoilage of shrimp. CT, *Clitoria ternatea* anthocyanin; G, gellan gum; HSPI, heat-treated soy protein isolate [73]. (**c**) RGB (red, green, blue) hue value changes of rose anthocyanin-containing films responding to increase in total volatile basic nitrogen (TVB-N) content during carp storage [51]. All figures were reprinted with permission from Elsevier.

The incorporation of different natural extracts into GG films has demonstrated significant potential in terms of developing freshness indicators for aquatic foods (Figure 12) (Table 6). For instance, the inclusion of *Clitoria ternatea* extract into GG films resulted in a noticeable color change from blue to bluish-green after 24 h of shrimp storage at room temperature [73]. This color transition was correlated with changes in the a* and b* color parameters and was linked to the increased TVB-N values, indicating the spoilage of the shrimp [73]. Similarly, black rice anthocyanin-incorporated GG films exhibited distinct color changes over time, shifting from red-orange on day 0 to brown-orange between days 1 and 3, green-yellow on day 4, and dark green by day 5 [41]. These color changes were directly associated with the freshness index of the shrimp, with red-orange indicating fresh shrimp, brown-orange indicating sub-fresh shrimp, and dark green signifying spoiled shrimp [41]. Elderberry anthocyanin-incorporated GG films also showed a significant color shift over time [95]. Initially mauve, the color lightened on the first day and eventually turned yellow-green as shrimp spoilage progressed. This change in transparency and color was visible to the naked eye and indicated the film’s responsiveness to variations in environmental pH values [95]. Similar results were observed for crab with mulberry anthocyanin-incorporated GG composites [96].

As for fish, the initial TVB-N value of fresh crucian fish was 4.7 mg/100 g, and the mulberry anthocyanin-integrated GG film was pink [33]. After approximately six days of storage, the TVB-N value increased to 20.7 mg/100 g, and the film color changed to light green. By the ninth day, the TVB-N value reached 40.7 mg/100 g, and the film color shifted to yellow-green, with corresponding RGB values reflecting these changes [33]. In another study, red radish anthocyanin-integrated GG films displayed a gradual color change during the spoilage of carp, transitioning from orange-red initially to green on day 7, and then to yellow-green by day 9 [44]. The B value decreased from 144 to 18 over this period, changing to a deeper yellow color, which serves as a characteristic parameter of the color change in indicator films [44]. Similar results were observed for black rice anthocyanin-integrated GG film that was used on largemouth bass [93].

These findings collectively highlight the potential of anthocyanin-incorporated GG films to act as visual indicators of seafood freshness. The color changes observed in these films are attributed to the pH-sensitive nature of anthocyanins and their interactions with volatile compounds produced during spoilage. The specific color transitions vary depending on the anthocyanin source and the type of seafood, but the overall trend of a color shift from red/orange to green/yellow appears to be consistent. Further research could focus on optimizing the sensitivity and specificity of these films for different aquatic species and storage conditions, as well as on exploring the potential to incorporate other pH-sensitive compounds or indicators.

#### 5.3.2. Intelligent Packaging for Other Animal-Derived Food Products

**Figure 13:**
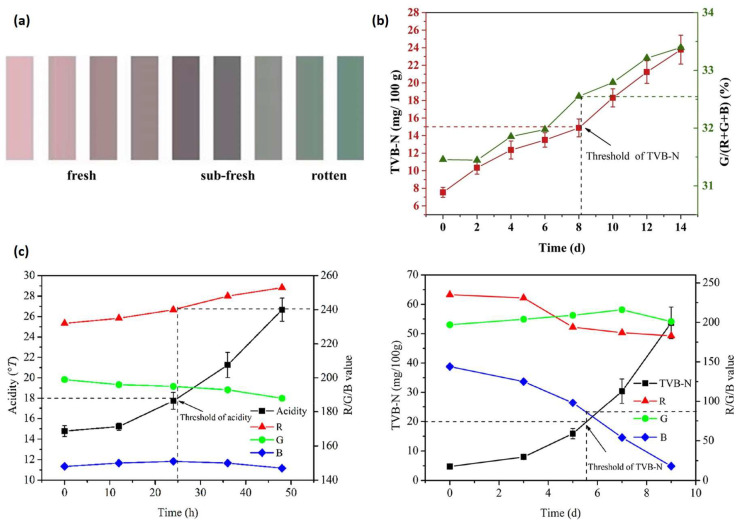
Freshness monitoring for other animal-derived food products. (**a**) Purple kale anthocyanin-containing film responding to chilled beef spoilage [94]. (**b**) RGB (red, green, blue) hue value changes of rose anthocyanin-containing films responding to the increase in total volatile basic nitrogen (TVB-N) content during chicken storage [51]. (**c**) RGB hue value changes in red radish anthocyanin-containing films responding to the increase in acidity and TVB-N during milk storage [44]. (**a**,**b**) were reprinted with permission from Elsevier. (**c**) was reprinted with permission from the American Chemical Society.

The effectiveness of GG-based freshness indicators extends beyond aquatic foods to various other animal-derived products (Figure 13) (Table 6). This versatility is demonstrated through studies on beef, chicken, pork, and milk, showcasing the broad applicability of these innovative indicators.

A study incorporating purple kale anthocyanin into GG films evaluated the freshness of beef using pH as an indicator [94]. The freshness was categorized into three stages: fresh (0–3 days), sub-fresh (4–6 days), and spoiled (after 7 days). The study found a positive correlation between the Δ*E* values of the indicator films and the TVB-N values of the beef, with correlation coefficients reaching up to 0.967 [94]. This high correlation suggests that the color changes in the GG films accurately reflect the spoilage level, making them reliable indicators of beef freshness.

For chicken, roselle anthocyanin-integrated GG films were utilized as freshness indicators [67]. The films changed color from pink on days 0–6 to reddish-brown on day 8 and to yellowish-brown on day 10, corresponding to the spoilage stages of chicken breasts. The Δ*E* values of the films increased over time, mirroring the color changes. The distinct color transitions from pink (fresh) to reddish-brown (sub-fresh) and yellowish-brown (spoiled) highlight the film’s effectiveness in monitoring chicken freshness [67].

In pork, rose anthocyanin-incorporated GG films demonstrated their utility by showing quantitative color changes over a 14-day storage period. The TVB-N values of pork samples increased from 7.54 to 23.78 mg/100 g. Concurrently, the color changes in the GG films were quantified using the ratio of the green (G) value to the sum of red (R), green (G), and blue (B) values (G/(R+G+B)). This ratio increased from 31.45% to 33.39%, indicating the sensitivity of the film to changes in pork freshness [51].

The application of GG films in monitoring milk spoilage was explored using red radish anthocyanin-incorporated GG films [44]. As the milk spoiled, the color of the indicator became redder, with the red (R) value increasing from 232 to 253, while the green (G) and blue (B) values remained relatively unchanged. This color change correlated with the increase in organic acid production during anaerobic bacterial respiration, as indicated by the rise in milk acidity from 14.78 °T to 25.67 °T after 48 h of storage at 25 °C [44]. The responsiveness of the film to acidity changes highlights its potential for monitoring milk spoilage.

In conclusion, GG-based freshness indicators hold significant promise for improving the safety and quality of a variety of animal-derived food products. Future research should focus on optimizing the formulation of these GG-based films for different food products and storage conditions.

#### 5.3.3. Intelligent Packaging for Fruits and Vegetables

**Figure 14:**
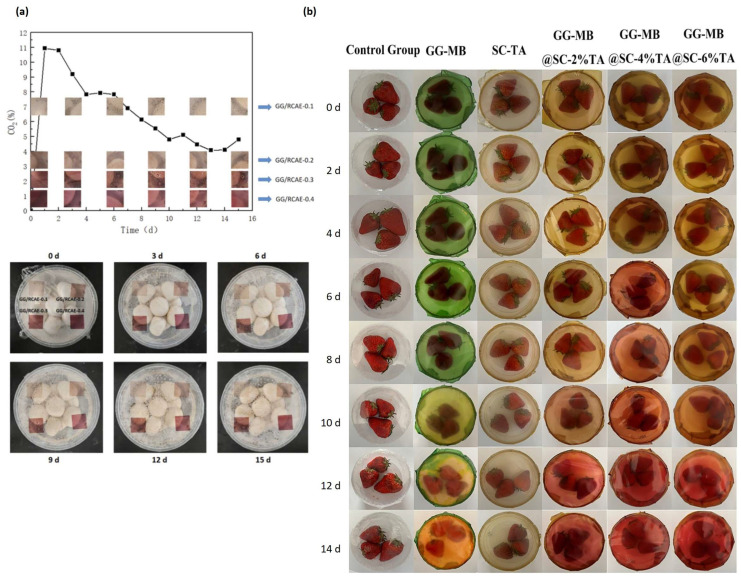
Freshness monitoring for fruits and vegetables. (**a**) Mushrooms protected with red cabbage extract-containing gellan gum film [43]. (**b**) Strawberries protected with methyl red/bromothymol blue-containing films. GG, gellan gum; MB, methyl red/bromothymol blue; SC, sodium carboxymethyl cellulose; TA, tannic acid [53]. Both figures were reprinted with permission from Elsevier.

The use of GG-based freshness indicators has also been explored for fruits and vegetables, demonstrating promising results in detecting spoilage and maintaining quality (Figure 14) (Table 6). In the case of mushrooms, red cabbage anthocyanin-integrated GG films were utilized to track freshness (Figure 14a) [43]. Before storage, the films exhibited a dark purplish-red color. As storage days increased, the surfaces of the mushrooms lost their luster and gradually turned yellow, indicating decreased freshness and the onset of spoilage. Correspondingly, the color of the GG film became brighter, changing from dark purple-red to light red. These color changes were easily observable by the naked eye, making the films effective indicators of mushroom freshness [43]. For strawberries, the incorporation of a methyl red/bromothymol blue into GG films provided a means to monitor spoilage (Figure 14b) [53]. During storage, the respiration of strawberries produced CO_2_, and the growth of bacteria and mold also resulted in the production of CO_2_, an acidic gas. This led to a reduction in pH within the GG film, causing a color change. Initially, the bilayer film was brownish-yellow from days 0 to 10, transitioning to reddish-brown between days 12 and 14, reflecting the spoilage process [53]. Future research could expand the application of GG-based freshness indicators to a broader range of fruits and vegetables. Studies could explore the effectiveness of different natural extracts and pH-sensitive compounds, optimize the formulation of GG films for various types of produce, and assess their performance under different storage conditions.

## 6. Conclusions

The study of GG-based composites for active and intelligent packaging has demonstrated their significant potential in enhancing food preservation and safety. GG, as a natural polysaccharide, provides a robust and adaptable matrix for the incorporation of various functional compounds, such as essential oils, anthocyanins, and nanoparticles. These additives enhance the mechanical, optical, antioxidant, and antimicrobial properties of GG-based composites, making them suitable for diverse applications in food packaging. The water vapor permeability and oxygen permeability of these composites can be either enhanced or inhibited with the addition of functional compounds, depending on the interaction between these substances and polymer matrices. The change in contact angle of these composites is reliant on the hydrophobicity of added ingredients. These active packaging materials can extend the shelf-life of plant-based food products and animal-derived products. The integration of colorimetric indicators enables the real-time monitoring of food freshness, offering a visual cue for quality assessment. Overall, GG-based films and coatings present an eco-friendly and effective solution for extending the shelf-life of perishable goods while reducing food waste. Further research is needed to optimize the formulation and scalability of these films and coatings to meet industry standards and consumer expectations.

## 7. Research Outlooks

The future of biodegradable active and intelligent packaging lies in the integration of advanced technologies and novel materials to further enhance functionality and consumer interaction. There are several future research directions that can be explored to better utilize GG in biodegradable active and intelligent packaging materials:

(1) One promising direction is the development of temperature-sensitive packaging systems that can provide visual or electronic alerts when products are exposed to unfavorable temperature conditions. This can be achieved through the incorporation of thermochromic dyes or thermos-responsive polymers that change color or opacity with temperature variations.

(2) Artificial intelligence (AI) is another frontier in intelligent packaging, enabling real-time data analysis and decision-making. AI algorithms can be embedded into packaging to monitor environmental conditions, predict shelf-life, and provide personalized usage recommendations to consumers. This technology can also facilitate supply chain optimization by tracking product history and ensuring quality control.

(3) The integration of smartphones into intelligent packaging offers a direct link between the product and the consumer. Near-field communication (NFC) or quick-response (QR) codes can be incorporated into packaging to provide instant access to product information, freshness indicators, and usage tips via mobile apps. This technology enhances consumer engagement and empowers consumers to make informed decisions about product consumption.

(4) In terms of materials, betalain-based dyes present an exciting opportunity for developing natural colorimetric indicators with broad pH sensitivity. These plant-derived pigments offer vibrant color transitions in response to pH changes, making them ideal for use in freshness indicators in food packaging. Moreover, betalains are known for their antioxidant properties, adding an additional layer of functionality to the packaging.

(5) The exploration of other functional compounds for active packaging is ongoing, with nanoparticles, phages, prebiotics, and probiotics offering promising results. Nanoparticles can enhance the barrier properties and mechanical strength of GG films, while phages provide targeted antimicrobial activity against specific pathogens. Probiotics can be incorporated into packaging to improve gut health and extend the shelf-life of fermented products.

(6) Other methods for the preparation of GG-based films and coatings should be investigated, such as 3D printing, extrusion, and electrospinning. The question of how to prepare these composites on an industrial scale should also be explored.

(7) Even though GG is considered a GRAS ingredient, further investigation into the safety of GG-based films and coatings is still necessary. This investigation should include cell-based methods and animal experiments.

In conclusion, the future of GG-based active and intelligent packaging is promising, with numerous opportunities for innovation and advancement. By combining the strengths of GG-based films with cutting-edge technologies and materials, the packaging industry can meet the evolving demands of consumers and contribute to a more sustainable and efficient food system.

## Figures and Tables

**Table 1:** Conditions to prepare gellan gum film.

Gel Base	Additives	Conditions	Refs.
GG up to 0.8%, levan up to 0.8%	Glycerol 0.4%, citric acid 0.04%	Polymers dissolved at 50 °C, dried at 50 °C overnight.	[12]
GG 2%, eggshell nanoparticles 1–3%	Glycerol 0.5%	Nanoparticles prepared by high-energy milling (2 h). Film solution cast and dried at 40 °C for 8 h.	[48]
GG 2%, polyvinyl alcohol 1%	Glycerol 30%, Caucasian whortleberry extract 0–9 mg/mL, myrtle essential oil 0–9 mg/mL	Solvent casting: polymers dissolved at 75 °C, additives incorporated, solution cast and dried at 35 °C for 24 h.	[9]
GG:κ-carrageenan:xanthan gum = 1:4:2	Not applicable	Polymers dissolved at 60 °C, cooled to room temperature, and cast and dried at 25 °C for 48 h.	[57]
GG:κ-carrageenan:xanthan gum = 2:6:1,	Glycerol 2.0 g, TiO_2_ 1–7%	Polymers dissolved at 80 °C, TiO_2_ added, cooled to room temperature, cast and dried at 25 °C for 48 h.	[34]
GG:Polyacrylamide:sodium citrate:sodium chloride:citric acid = 3:3:3:2:2,	Glycerol 2.0 g, ZnO 1–5%	Polymers dissolved at 80 °C, ZnO added, solution cast and dried at 25 °C for 72 h.	[11]
GG 0.2%, citrus pectin 1%	Glycerol 0.5%, CaCl_2_ 5 mM, EDTA 0.05 M, antimicrobial supernatant 50 AU/mL	Polymers dissolved at 75 °C, additives incorporated, solution cast and dried at 35 °C for 17 h, conditioned at 23 °C for 48 h.	[42]
GG 1%, agar 1%, montmorillonite clay 0–10%	Glycerol 1%, CaCl_2_	Polymers dissolved at 80 °C, clay added, solution cast and dried at 25 °C for 48 h.	[52]
GG 1%, black rice extracts 0–12%	Glycerol 1%	GG dissolved at 50 °C, extracts added, solution cast and dried at 40 °C for 5 h.	[93]
GG 1%, alginate 1%, ZnO 5%,	Glycerol 0.8%, black rice anthocyanin, and curcumin	GG layer cast first, GG dissolved at 60 °C, anthocyanin, and curcumin added, alginate-ZnO layer casted afterward and dried at 25 °C for 60 h.	[41]
GG 1%, CMC, starch 2%,	Glycerol 0.8%, roselle anthocyanin, nisin	GG dissolved at 70 °C, cooled to 40 °C, roselle anthocyanin added, casting for outer layer with CMC, starch, and nisin	[67]
GG 0.5%, apple pectin 2%	Glycerol 2.2%, nisaplin	Polymers dissolved at 70 °C, nisaplin added, solution cast and dried at 35 °C for 11 h.	[65]
GG 1%, Chitosan 2%	Glycerol 0.25%, thyme essential oil emulsion 2–6%	GG layer cast first, polymers dissolved at 70 °C, dried at room temperature for 4 h, followed by chitosan–nanoemulsion layer containing essential oil emulsion, dried at room temperature for 12 h	[58]
GG 0.7%, xanthan gum 0.3%, alginate 0.7%, hydroxypropyl methylcellulose 0.7%	Glycerol, beeswax, fructose, sorbitol, or polyethylene glycol, 1%	Polymers dissolved at 50 °C, cooled, cast and dried at 28 °C for 3 h	[98]

Abbreviation: CMC, carboxymethyl cellulose; EDTA, ethylene diamine tetraacetic acid; GG, gellan gum; PVA, polyvinyl alcohol.

**Table 2:** Mechanical properties of gellan gum-based composites.

Functional Compounds	Gel Base	Tensile Strength (MPa)	Elongation at Break (%)	Refs.
TiO_2_ nanoparticles	GG	↑ from 48.2 to 56.1	↓ from 28.2 to 23.0	[34]
TiO_2_ nanoparticles	GG, carrageenan, xanthan gum	↑ from 40.5 to 56.1	↓ from 30.6 to 23.0	[34]
ZnO nanoparticles	GG/xanthan gum	↑ from 22.1 to 35.5	↓ from 30 to 25.1	[77]
ZnO nanoparticles	GG	↑ from 33.5 to 43.8	↑ from 24.6 to 32.8	[34]
SiO_2_ nanoparticles	GG/cellulose	↑ from 28.2 to 45.1	↓ from 23.1 to 34.2	[89]
Eggshell nanoparticles	GG	↓ from 73.79 to 54.93	↓ from 36.7 to 25.4	[48]
Black rice anthocyanins	GG/zein	↑ from 7.58 to 8.91	↓ from 7.68 to 5.37	[93]
Elderberry anthocyanins	GG/gelatin	↑ from 5.46 to 14.57	No influence (17.92–18.59)	[95]
*Clitoria ternatea* anthocyanins	GG	No influence (~11)	No influence (~6)	[73]
Rosemary essential oil	GG/cellulose	↓ from 14.56 to 8.63	↓ from 12.13 to 7.78	[78]
Dill essential oil	GG/cellulose	↑ from 5.82 to 10.39	↓ from 95.82 to 78.87	[13]
Cranberry extract, *Lactococcus lactis*	GG	↑ from 17.92 to 32.52	↑ from 12.32 to 19.23	[30]
*Anethum graveolens* essential oil	GG/cellulose	↑ from 5.82 to 10.36	↓ from 95.82 to 78.87	[13]

Abbreviation: GG, gellan gum; Note: ↑, promotive effect; ↓, inhibitory effect.

**Table 3:** Antimicrobial effects of gellan gum-based composites.

Functional Compounds	Gel Base	Methods	Major Results	Refs.
ZnO	GG	Antibacterial: *Salmonella enterica*, *Bacillus cereus*, *Staphylococcus aureus*, *Cronobacter*, *sakazakii*, *Escherichia coli*	1% ZnO showed no antibacterial effect *S. aureus* was the most susceptible to the films *B. cereus* was most resistant to the films	[11]
TiO_2_ nanoparticles	GG, carrageenan, xanthan gum	Antibacterial: *Pseudomonas aeruginosa*, *S. aureus*, *Acinetobacter baumannii*, *E. coli*	3% TiO_2_ showed no antibacterial effect 5–7% TiO_2_ showed antibacterial effects	[34]
TiO_2_ nanoparticles	GG	Antibacterial: *S. aureus*, *Streptococcus*, *E. coli*, and *P. aeruginosa*	The inhibition zone was comparable with the penicillin control sample.	[35]
Silver nanoparticles	GG	Antifungal: *Candida* spp. (*C. albicans*, *C. lusitaniae*, *C. haemulonii*, *C. krusei*, *C. glabrata*)	All the fungal species were sensitive to Ag nanoparticles. The minimum required concentration of Ag for inhibiting the growth of all fungal species was 0.063 mg/mL.	[88]
SiO_2_ nanoparticles	GG, cellulose	Antibacterial: *B. cereus*, *E. coli*, S. aureus, *Cronobacter sakazakii*, *Salmonella enterica*, *Salmonella* Typhimurium	↑ Antimicrobial effects, with a dose-dependent manner. The film was more active against *S. aureus* and less active against *B. cereus.*	[89]
Coffee parchment waste	GG	Antifungal: *Fusarium verticillioides*, *Fusarium* sp., *Colletotrichum gloeosporioides*	Blanks did not show growth inhibition. The presence of caffeine and phenolic compounds in the films was beneficial for acquiring natural antifungal properties.	[36]
Mustard essential oil	GG, chitosan	Antibacterial: *E. coli*, *S. aureus*, *B. anthracis*	↑ Antimicrobial effects with a dose-dependent manner. Sensibility: *B. anthracis* > *S. aureus* > *E. coli*	[38]
Rosemary essential oil	GG, cellulose	Antibacterial: *E. coli*, *S. aureus*, *Salmonella* Typhimurium, *P. fluorescence*	Inhibitory effects increased with the increase in concentration. *P. fluorescence* showed the lowest sensitivity.	[78]
Thyme essential oil	GG, starch	Antifungal: *B. cinerea*	No inhibitory effect was observed on an inoculated apple.	[104]
Nisin	GG, guar gum	Antibacterial: *B. subtilis*, *E. coli* Antifungal: *Saccharomyces cerevisiae*	Films showed more effective antimicrobial activity against *B. subtilis* than *E. coli* The inhibitory effect against *S. cerevisiae* was not as apparent as that against the other bacteria.	[60]
Nisin	GG, pectin	Antibacterial: *L. monocytogenes*	A neat film and a film containing (129.7 IU/mL) nisin did not prevent the growth. The minimum inhibitory concentration of nisin was 171.5 IU/mL.	[65]
Three different natural polycationic polymers	GG, chitosan	Antibacterial: *S. enteritidis*, *S. aureus*	*S. aureus* cells attach more to the film surfaces than the *S. enteritidis.* ↑ Antimicrobial effects	[56]
Not applicable	Oxidized GG	Antibacterial: *E. coli*, *S. aureus* Antifungal: *Aspergillus niger*	Antibacterial and antifungal activity was improved with an increasing oxidation level	[102]

Abbreviation: GG, gellan gum. Note: ↑, promotive effect; ↓, inhibitory effect.

**Table 4:** Preservation of plant-based food products with gellan gum-based composites.

Functional Compounds	Gel Base	Food	Major Results	Refs.
Thyme essential oil	GG, starch	Apples and persimmons stored at 25 °C for 14 d	↓ Weight loss, ↑ firmness maintenance, ↓ respiration rate, ↓ severity of gray mold	[104]
Geraniol and pomegranate extract	GG	Fresh-cut strawberries stored at 5 °C for 7 d	↓ Microbial growth, ↑ firmness maintenance, ↓ off-odor	[86]
Thymol–β-cyclodextrin microcapsules	GG, konjac glucomannan	Blueberries stored at 2 °C for 56 d	↓ Decay rate, ↓ weight loss, ↓ respiration rate, ↓ softening and senescence, ↑ cuticular waxes, ↓ lipid oxidation, ↓ terpenes, ↓ benzaldehyde, ↑ esters, ↑ aldehydes	[84]
*Lactococcus lactis* and cranberry extract	GG	Fresh-cut apples and potatoes stored at 4 °C for 10 d	↓ Browning, ↑ firmness maintenance, ↑ antioxidant activity	[30]
Oregano essential oil	GG	Mandarin stored at 6 °C for 24 d and at 15 °C for 7 d	↓ Weight loss, ↑ firmness maintenance, ↑ color preservation, ↑ total phenolic and content, ↑ antioxidant activity ↑ ascorbic acid maintenance, ↓ microbial growth	[39]
Ascorbic acid	GG	Litchi stored at 5 °C for 7–14 d	↓ Weight loss, ↑ color maintenance, ↑total soluble solids preservation	[109]
Vanillin and geraniol	GG, apple fiber	Banana stored at 5 °C for 12 d	↑ Firmness maintenance, ↑ color maintenance, ↑ antioxidant activity, ↓ yeasts/molds growth, ↑ sensory shelf-life by 4 d	[83]
Not applicable	GG, chitosan, alginate	Apricot stored at 4 °C and 80% RH for 15 d	↑ Ascorbic maintenance, ↑ external color maintenance, ↑ carotenoids maintenance, ↓ weight loss, ↑ firmness maintenance, ↑ firmness maintenance, ↓ peroxidase, ↓ polyphenol oxidase	[8]
Mustard essential oil	GG, chitosan	Mango stored at 25 °C and 80% RH for 20 d	↓ Total soluble solids increase, ↓ titratable acidity decrease, ↑ vitamin C maintenance, ↓ decay rate, ↑ firmness maintenance	[38]
Not applicable	GG, chitosan, lactoferrin	Strawberry stored at 25 °C and 50% RH for 6 d	↓ Weight loss, ↓ titratable acidity decrease, ↓ total soluble solids increase, ↓ microbial growth	[70]
1-MCP	GG	Jackfruit bulbs stored at 5 °C for 14 d	↓ Total soluble solids increase, ↓ titratable acidity decrease, ↓ respiration rate, ↑ firmness maintenance, ↓ microbial growth	[108]
*Anethum graveolens* essential oil	GG, pineapple peel cellulose	Bread stored at room temperature for 3 weeks	↓ Microbial growth	[13]
Natamycin, essential clove oil	GG, citrus pectin	Tortilla stored at 22 °C and 50% RH for 30 d	Sample with a high density of polyethylene exhibited a sour odor. The active film did not negatively affect the sensory attributes of the food.	[64]
Not applicable	GG, waxy corn starch	Rice cakes stored at 25 °C with 45% RH for 4 d	↓ Retrogradation, ↑ moisture maintenance (3.36% for uncoated sample vs. 1.08% for coated sample), ↑ texture profile	[111]

Abbreviations: 1-MCP, 1-methylcyclopropene; GG, gellan gum; RH, relative humidity. Note: ↑, promotive effect; ↓, inhibitory effect.

**Table 5:** Preservation of animal-derived products with gellan gum-based composites.

Functional Compounds	Gel Base	Food	Major Results	Refs.
Polyester, phenolic acids	GG, starch	Pork stored at 5 °C and 48% RH for15 d	↓ pH decrease, ↓ TBARS, ↓ TVB-N, ↓ microbial growth, ↓ weight loss, ↓peroxide index, ↓ Δ*E* of meat	[26]
*Aronia melanocarpa* extract	GG, pea protein, chitosan	Pork stored at 4 °C for 7 d	↓ TVB-N, ↓ weight loss (20% for active film-protected sample vs. 37.72 for unwrapped sample),	[72]
*Alhagi sparsifolia* flower extract	GG, PVA	Dried shrimp stored at 37 °C for 40 d	↓ TBARS value by 47.5%, ↓ peroxide index, ↓ protein oxidation. Bilayer films were more effective than single-layer film.	[74]
Not applicable	GG, konjac glucomannan	Snakehead fillets stored at −20 °C for 150 d	↑ Hardness, ↑ springiness, ↑ chewiness, ↓ TVB-N, ↓ TBARS	[62]
Anthocyanin, nisin	GG, cellulose, starch	Chicken breast stored at 4 °C for 10 d	↓ TBARS, ↑shelf-life of by 1–2 d, ↓ pH increase	[67]
Caucasian whortleberry extract, myrtle essential oil	GG, PVA	Turkey breast stored at 4 °C for 15 d	↓ TVB-N, ↓ peroxide value, ↓ TBARS, ↓ pH decrease, ↑ sensory properties	[9]
Basil essential oil	GG	Egg stored at 25 °C for 42 d	↓ Weight loss, ↑ Haugh unit, ↑ yolk index, ↓ total aerobic plate count, ↑ pH maintenance	[80]
Potassium sorbate	GG, aloe gel	Cheese stored at 25 °C for 12 d	↓ Weight loss, ↓ growth of *Penicillium roqueforti*	[29]

Abbreviations: GG, gellan gum; PVA, polyvinyl alcohol; TBARS, thiobarbituric acid; TVB-N, total volatile base nitrogen. Note: ↑, promotive effect; ↓, inhibitory effect.

**Table 6:** Intelligent packaging of gellan gum-based composites.

Dye and Gel Base	Gel base	Colorimetric Response	Application	Major Results	Refs.
Black rice extracts	GG and zein	Red (pH 2) to pale pink (pH 4–6), purple (pH 8–10) and finally taupe (pH 11–12)	Largemouth bass fillets stored at 4 °C for 10 d	The Δ*E* of film at 2–6 d ranged from 6.83 to 9.08. Pink to brown (8 d, Δ*E* = 13.14, spoilage point). Grayish-brown (10 d, rotten)	[93]
Roselle anthocyanins	GG, carboxymethyl cellulose, starch	Red (pH 2–3), dark pink (pH 4), red (pH 5–6), light pink (pH 7), red (pH 8), dark green (pH 12), and yellow-green (pH 13)	Chicken stored at 4 °C for 10 d	The bilayer films were pink on days 0–6, reddish-brown on days 8 and yellowish-brown on day 10 Extended chicken shelf-life by 2 d	[67]
Methyl red, bromothymol blue	GG, cellulose	Orange → pink → yellow → green → blue trend at different pH conditions	Lamb meat stored at 4 °C for 7 d	From orange-red on 0 d, orange-yellow on 1–4 d, yellow-green on 5–6 d, to green on 7 d The values of TVC, pH and TVB-N were positively correlated with each other	[114]
Black rice anthocyanins and curcumin	GG	Orange-red → orange → yellow → gray-yellow → red-brown → black-red → bright red as the pH went from 2 to 12	Not applicable	Not applicable	[49]
Red radish anthocyanins	GG, gelatin	From deep orange red to light carmine (pH range of 2–7), purple (pH 8–9), yellow-green (pH 10), yellow (pH 11–12)	Milk placed in an incubator at 25 °C with 75% RH for 48 h. Black carp placed in an incubator at 4 °C with 75% RH for 10 d	Milk: The acidity of milk showed 18 °T at 25 h, R value of the film was ∼240 Carp: color of film changed color from orange-red to green (7 d), followed by yellow-green. TVB-N value showed 20 mg/100 g at 5.5 d, and B value of film showed ∼87.	[44]
Mulberry anthocyanins	GG, gelatin	From light pink to colorless (pH 2–6), from light green to yellowgreen (pH 7–10), orange color (pH 11–12)	Crucian carp at 4 °C with 75% RH for 9 d	Color of film changed from pink to light green (6 d). TVB-N value increased from 4.7 to 20.7 mg/100 g (6 d)	[33]
*Clitoria ternatea* extract	GG, heat-treated soy protein isolate	Red (pH < 3), violet (pH 3.0–5.0), blue (pH 5.0–6.0), blue-green (pH 7.0–9.0), green (pH 10.0–11.0) and brownish-yellow (pH > 11.0)	Shrimp at room temperature (25 °C) for 24 h	Colors of films changed from blue (initial) to bluish-green Δ*E* of films was correlated with the increase in TVB-N values	[73]
Broussonetia papyrifera fruit anthocyanin	GG, mica nanosheets, konjac glucomannan, carrageenan	Crimson (pH 2–3), light red and almost colorless (pH 4–6), blue-grey (pH 7–11), yellow (pH 12)	Shrimp at 4 °C for 5 d and 25 °C for 15 h	25 °C: color of films showed no changes in the first 6 h. Color of films changed from purple to yellow/blue at 9 h 4 °C: pH and TVB-N increased to 7.45 and 29.33 g/100 g after 3 d. Δ*E* of films responded well to shrimp spoilage	[55]
Elderberry anthocyanins	GG, guar gum	Color shifted from red to pink, lavender to purple, and dark purple to yellow-brown as the pH went from 2 to 12	Shrimp stored in a 4 °C for 4 d	The color of the film changed from mauve to lighter on 1 d, and eventually showed a yellowish-green. Color change responds well to variations in pH. Anthocyanin layer inhibited protein degradation	[95]
Mulberry anthocyanin	GG, chitosan, polyvinyl alcohol, alginate	Not applicable	Chinese mitten crab stored at 4 °C for 10 d	The color of films showed a visible change from pink to dark green, correlated with the TVB-N level (increased to 31.23 mg/100 g on day 8).	[96]
Methyl red, bromothymol blue	GG, alginate, cellulose, tannic acid	A consistent dark red tint, turning brown-red (pH 4–5), followed by gray-green (pH 6–8), and ultimately a deep green (pH 9–10).	Strawberry stored at 4 °C for 14 d	Δ*E* change in the composite film containing 6% tannic acid was the largest. Double-layer film delayed the rotting and deterioration of strawberry.	[53]
Red cabbage anthocyanins	GG	Red (pH 2), pink (pH 3), purplish-red (pH 4–6), purple (pH 7), blue (pH 8–9), green (pH 10)	Mushroom stored at 4 °C for 15 d	The color of films was dark purplish-red before storage, and it changed to light red during storage. The surface of mushroom lost its luster and gradually turned yellow in color.	[43]

Abbreviations: GG, gellan gum; RH, relative humidity; TVB-N, total volatile basic nitrogen; TVC, total viable count; Δ*E*, change in color.
